# Feasibility of Gamified EEG Neurofeedback Combined with Adaptive Working-Memory Training in Older Adults: A Pilot Study

**DOI:** 10.3390/brainsci16080865

**Published:** 2026-08-15

**Authors:** Ping-Chen Tsai, Kea-Tiong Tang, Asangaedem Akpan, Heba Lakany

**Affiliations:** 1 Department of Electrical Engineering and Electronics, University of Liverpool, Liverpool L69 3GJ, UK; pingchen@liverpool.ac.uk; 2 Department of Electrical Engineering, National Tsing Hua University, Hsinchu 30013, Taiwan; kttang@mx.nthu.edu.tw; 3 Department of Geriatric Medicine, Bunbury Regional Hospital, Western Australia Country Health Service South West, Bunbury, WA 6231, Australia; asan.akpan@health.wa.gov.au; 4 Division of Internal Medicine, University of Western Australia, Perth, WA 6009, Australia; 5 Medical School, Faculty of Health Sciences, Curtin University, Perth, WA 6102, Australia

**Keywords:** brain–computer interface, cognitive augmentation, neurofeedback, working memory, inhibitory control, balance, ageing, fall prevention

## Abstract

**Highlights:**

**What are the main findings?**

- A 24-session gamified EEG neurofeedback programme combined with an adaptive N-back task was feasible and well tolerated in a supervised experimental setting in community-dwelling older adults, with complete adherence.
- Left-frontal alpha power rose at the trained neurofeedback site, frontal theta increased bilaterally under working memory load, and scores changed in the direction of improvement on balance, gait, and cognitive measures, although functional changes were small, and cognitive changes cannot be separated from repeated-testing effects.

**What are the implications of the main findings?**

- At the participant level, right-frontal alpha desynchronisation tracked balance and gait adaptability, and bilateral frontal theta tracked processing speed—an exploratory shortlist of candidate features linking the trained rhythms to functional mobility.
- These features, together with their effect-size estimates, provide the basis for powering a sham-controlled trial in a fall-prone population.

**Abstract:**

Background: Falls among older adults are linked to central nervous system deterioration and executive function deficits. Objectives: This study evaluates the feasibility of a gamified EEG-based neurofeedback intervention combined with adaptive working-memory training, targeting neural processes associated with attentional inhibition and working memory in older adults, and characterises, as exploratory secondary outcomes, the oscillatory and functional measures that change over the training period. Methods: Twenty healthy older adults (65.0±3.3 years) completed a 24-session longitudinal training programme. The intervention combined real-time alpha-band neurofeedback (NF), targeting left-frontal alpha activity associated with inhibitory control, with an adaptive N-back task engaging theta-band working memory processes. Behavioural outcomes included the Colour Trail Making Test A and B (CTMT-A/B), the Berg Balance Scale short form (BBS-3P), and the four-item Dynamic Gait Index (DGI-4). EEG was recorded at the Early, Middle, and Later stages to characterise training-related neural and behavioural changes. Results: The programme proved highly deliverable: adherence was 100% across all 24 sessions, no session was terminated early, and no severe adverse events occurred. Participants acquired control of the trained signal, with left-frontal alpha power rising across training at the neurofeedback target site, and frontal theta increased bilaterally under adaptive working memory load, consistent with the reduced hemispheric asymmetry characteristic of this age group. Scores changed in the direction of improvement on all four behavioural measures; however, functional changes were small, and cognitive changes cannot be separated from repeated-testing effects. Of sixteen candidate EEG–behaviour associations, four showed moderate-to-large participant-level coefficients and were retained as candidate associations: right-frontal alpha with balance (r=−0.64) and with gait adaptability (r=−0.48), and bilateral frontal theta with processing speed (r=−0.53 and −0.51). Several associations that appeared strong when observations were pooled across stages did not survive participant-level modelling, indicating that repeated-measures methods are needed in this literature. All associations are exploratory, uncorrected for multiplicity, and reported with effect sizes and confidence intervals. Conclusions: The gamified neurofeedback and working-memory training programme was feasible, well tolerated, and fully adhered to in a supervised experimental setting by community-dwelling older adults and was accompanied by measurable modulation of the targeted frontal rhythms. This study delivers a shortlist of candidate EEG features, with the effect-size estimates needed to power a confirmatory trial. Because the design is single-arm, both functional scales approached ceiling, and the same test forms were repeated at each stage, practice effects cannot be separated from intervention effects, and these associations are hypothesis-generating; a sham-controlled trial in a fall-prone population is the appropriate next step.

## 1. Introduction

The increasing prevalence of falls among the global geriatric population represents a critical challenge that intersects epidemiology and neurophysiology [1,2]. While traditional clinical paradigms have largely attributed risk of fall to peripheral physical decline [3], such as sarcopenia or vestibular degradation, emerging evidence implicates the deterioration of the central nervous system as a primary driver of gait and balance instability [4]. Safe locomotion in complex environments is not merely a motor reflex but a cognitively demanding executive task requiring continuous monitoring and rapid decision-making [5,6]. Recent longitudinal research has fundamentally recalibrated this understanding, demonstrating that deficits in executive function serve as reliable early predictors of falls, often predicting risk more accurately than global measures of cognitive decline [7]. Furthermore, recent studies have demonstrated that integrated motor–cognitive training, such as exergaming, can successfully modulate prefrontal cortex activity and improve gait parameters in healthy older adults, suggesting a direct link between enhanced executive control and motor stability [8,9,10]. Within this executive hierarchy, working memory provides an interface between high-level goal selection and ongoing action by maintaining and updating information required for adaptive locomotion [11,12,13]. On the other hand, the fidelity of working memory processes relies on inhibitory control to maximise the signal-to-noise ratio by filtering out task-irrelevant neural activity [14,15,16]. However, ageing is associated with a specific degradation in this selective inhibition, allowing distractor stimuli to consume limited processing bandwidth and destabilise gait [17]. Consequently, there is a critical need to expand prevention strategies beyond resource-intensive clinical therapies. Unlike physical therapy, which typically addresses established functional decline, these neurocognitive interventions can proactively target latent executive deficits, potentially mitigating the risk of fall before the onset of overt motor deterioration.

Electroencephalography (EEG) provides a non-invasive, temporally precise, and relatively inexpensive method for assessing neurophysiological function. Building on this capability, Brain–Computer Interfaces (BCIs) have emerged as a powerful tool for cognitive and motor rehabilitation. A recent systematic review has highlighted that while EEG-based BCI interventions show significant promise for enhancing cognitive functions such as working memory and attention in older adults, their translation to clinical rehabilitation is currently hindered by methodological heterogeneity and a lack of robust evidence regarding the transfer of these cognitive gains to physical function recovery [18]. Neurofeedback (NF), a core paradigm within these systems, relies on operant conditioning [19] and Hebbian plasticity [20,21] to promote the adaptive neural changes necessary for such transfer.

Complementing this direct neural training, standardised cognitive assessments such as the N-back task are widely utilised to challenge and enhance working memory [22,23]. By requiring the continuous monitoring and updating of incoming information, these tasks directly engage executive function networks. Recent electrophysiological studies confirm that neural activity recorded during N-back performance provides a reliable metric of working memory load and neural efficiency [24,25]. These neural signatures provide a means of monitoring training-related plasticity and the transfer of cognitive gains [26], while also elucidating the specific and often altered neural recruitment strategies employed by older adults [27]. Therefore, integrating working memory loads into a neurofeedback protocol creates a dual-process training environment that targets the cognitive–motor interface essential for fall prevention.

This study targets two specific neural oscillations in training these executive capacities: alpha (8–13 Hz) and theta (4–7 Hz) rhythms.

Frontal alpha oscillations are no longer viewed as passive idling rhythms but are now understood to mediate active inhibitory control. As described by the inhibition-timing hypothesis, high-amplitude alpha activity functions to suppress task-irrelevant neural populations, effectively gating out distractions to protect information processing [28]. In the context of motor control, the modulation of alpha oscillations around the sensorimotor area follows a distinct pattern. Specifically, Event-Related Synchronisation (ERS) involves increased power, which reflects cortical inhibition or an idling state. In contrast, Event-Related Desynchronisation (ERD) is characterised by a decrease in power, marking the active engagement of cortical areas during movement planning [29]. In the context of attentional inhibition, increased frontal and prefrontal alpha power has been consistently linked to the successful inhibition of distractors and the protection of working memory retention intervals [30,31]. For instance, studies using spatial cueing tasks demonstrate that alpha power increases ipsilateral to the attended hemifield, effectively ’gating out’ competing visual information to facilitate target selection. BCI protocols have successfully trained users to modulate alpha activity to enhance these inhibitory capacities. For example, neurofeedback training targeting alpha up-regulation has been shown to improve cognitive performance [32,33,34]. Thus, by reinforcing alpha oscillations, it is possible to potentiate inhibitory control, allowing for more robust gating of distractors and the preservation of cognitive stability.

Concurrently, frontal theta oscillations serve as a reliable marker of working memory, scaling with cognitive load [35,36]. Building on this, recent evidence identifies mid-frontal theta synchronisation as the primary neurophysiological signature of the executive recruitment necessary for complex motor planning [37]. This oscillatory increase over frontoparietal regions mediates top-down control, supporting the selection and sequencing of motor commands [38]. Notably, older adults often show reduced theta power or even ERD, indicating a failure to recruit the executive resources required for the task [39,40]. This neural inefficiency reflects an inability to sustain the working memory load required for stable coordination, identifying frontal theta modulation as a candidate neural index relevant to cognitive–motor function in ageing [41]. Thus, analysing theta dynamics offers valuable insights into the neural substrates of working memory and the associated neural inefficiency.

Two features of the ageing brain bear directly on how these rhythms should be measured. Frontal recruitment becomes less lateralised, with older adults engaging bilateral regions for tasks that are lateralised in younger adults [42] and showing expanded, bilaterally symmetric ERD in the sensorimotor domain [43]. Any frontal feature used to index training in this population should therefore be extracted from both hemispheres rather than assumed to be left-lateralised.

The relevance of these rhythms to mobility is established observationally. Older adults recruit prefrontal resources to sustain motor performance as subcortical control declines [44], and this recruitment is visible as expanded sensorimotor desynchronisation [43]. In the same population, higher resting theta power is associated with better attention and executive function [45], while the capacity to modulate frontal theta with task demand is itself attenuated in ageing [39,46]. Alpha and theta are therefore not interchangeable indices: the first tracks how strongly cortex is engaged by a postural or attentional demand, and the second tracks how much executive resources are recruited to meet it.

Neurofeedback offers a route to modulating the first of these directly. Training older adults to up-regulate frontal alpha has been shown to improve cognitive performance [32,33,34], but whether such training reaches balance and gait remains largely untested: the transfer step from a trained oscillatory feature to functional mobility is the specific gap this study addresses [18].

The present study is a single-arm feasibility study of an accessible, gamified EEG-based intervention combining real-time alpha-band neurofeedback with an adaptive N-back working memory task in community-dwelling older adults. Its purpose is to establish whether the programme can be delivered and tolerated in this population and to characterise which oscillatory features move over the course of training and how they relate to cognitive and functional performance. It is not designed to demonstrate that the intervention causes those changes.

Based on this framework, we formulated three hypotheses.

1. Trained inhibitory gating.Because the neurofeedback contingency reinforces alpha up-regulation at a left-frontal site, left-frontal alpha power should increase across the programme, consistent with strengthened inhibitory gating [28,30]. Under the same framework, alpha desynchronisation elsewhere would index active cortical engagement rather than a failure of the training [29].
2. Working memory engagement and its spatial profile. Adaptive N-back load should engage frontal theta [47]. Because frontal recruitment in older adults is characteristically less lateralised than in younger adults [42,43,48], we expect this engagement to be bilateral rather than confined to the left hemisphere. We do not treat CRUNCH [49] as a testable prediction here, since the present design does not manipulate load independently of training.
3. Exploratory cognitive–motor coupling. If executive processes are shared between working memory and postural control, the oscillatory features that track the trained processes should co-vary with balance, gait, and cognitive performance at the participant level. This hypothesis is exploratory: this study is not powered to test it, has no comparator, and any association observed is compatible with, but cannot establish, transfer.

## 2. Methods

### 2.1. Participants

**Figure 1:**
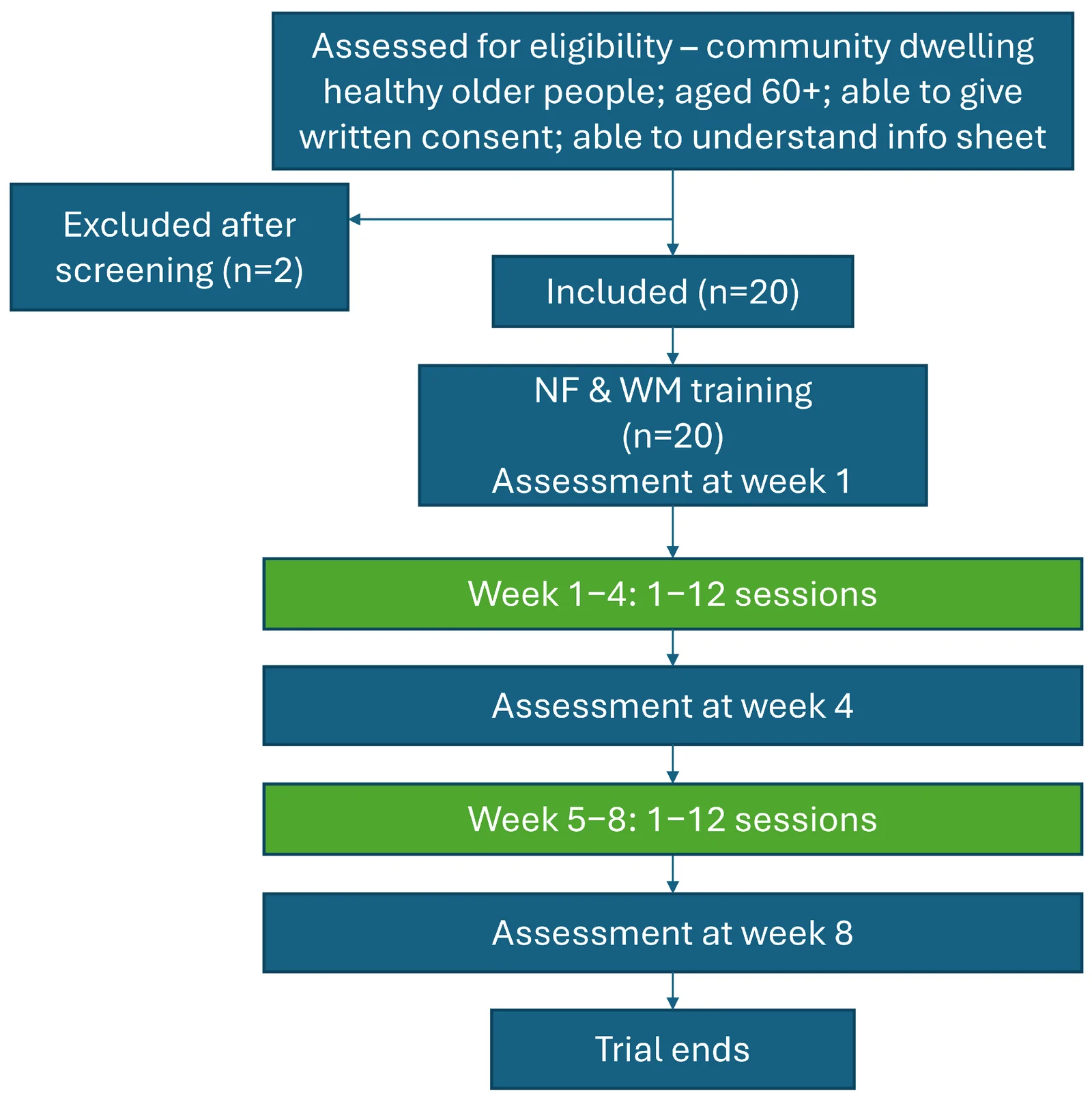
Flow diagram of the study design. The chart illustrates participant screening and enrolment (*n* = 20), the combined neurofeedback (NF) and working memory (WM) training schedule over 24 sessions, and the timing of outcome assessments at Weeks 1, 4, and 8. Arrows indicate the flow of participants through the study; blue boxes denote the screening, enrolment, and assessment steps, and green boxes denote the two blocks of training sessions.

Twenty community-dwelling older adults (13 female, 7 male; 65.0±3.3 years) completed an 8-week programme consisting of 24 sessions as shown in Figure 1. All participants were healthy, right-handed, and had no history of neurological or psychiatric disorders (e.g., epilepsy, stroke, traumatic brain injury, major depression, or psychosis). To ensure normal cognitive function, the Mini-Mental State Examination (MMSE) [50] was administered, yielding a mean score of 29.2±0.5; all participants scored above the normal threshold of >25. Each session included two modules: (i) alpha-band adaptive neurofeedback (NF) for attentional inhibition and (ii) an adaptive N-back task for working memory. Participants abstained from alcohol for 24 h before testing and were asked to abstain from nicotine and from caffeine-containing beverages for at least 1 h before the experiment. The study was approved by National Tsing Hua University Research Ethics Review Committee (11201HE007), and all participants gave their written informed consent. Behavioural and EEG measures were recorded throughout. Ethics approvals and written informed consent were obtained prior to participation.

#### Session-Day Exclusions and Withdrawal

On any given visit, data collection was deferred if participants reported acute illness, fever, or recent alcohol/recreational drug use; participants were asked to avoid unusually high caffeine intake and to maintain regular sleep prior to testing. Participants could withdraw at any time. The investigator could discontinue participation in cases of adverse events (e.g., persistent headache/dizziness), inability to tolerate the EEG cap or task demands, poor EEG signal quality across multiple sessions despite remediation, or repeated non-adherence (e.g., missing >20% of scheduled sessions).

### 2.2. Experimental Design

This study was a prospective, longitudinal, single-arm trial (Figure 1). Twenty community-dwelling healthy older adults completed a combined neurofeedback and working-memory training protocol. Each training session combined adaptive neurofeedback (NF) with an adaptive N-back working memory (WM) task. All participants received the same intervention; no parallel control group was implemented. The design reflects the feasibility and mechanistic focus of this early-phase investigation.

#### Training Schedule

Participants completed 24 sessions of training over 8 weeks (3 sessions per week). Each session followed a fixed sequence: (1) setup (10 min), (2) NF module (15 min), and (3) adaptive N-back module (20 min). The NF-to-N-back order was held constant across sessions to minimise order effects.

Clinical and cognitive outcome measures, as detailed in Section 2.7, were obtained at three predefined time points: baseline prior to the first intervention Week 1, mid-intervention at Week 4, and post-intervention at Week 8. These assessments were aligned with the beginning, mid-point, and completion of the 24-session NF+WM training programme in order to characterise trajectories of motor and cognitive change over the course of the intervention.

### 2.3. Apparatus and Setup

**Figure 2:**
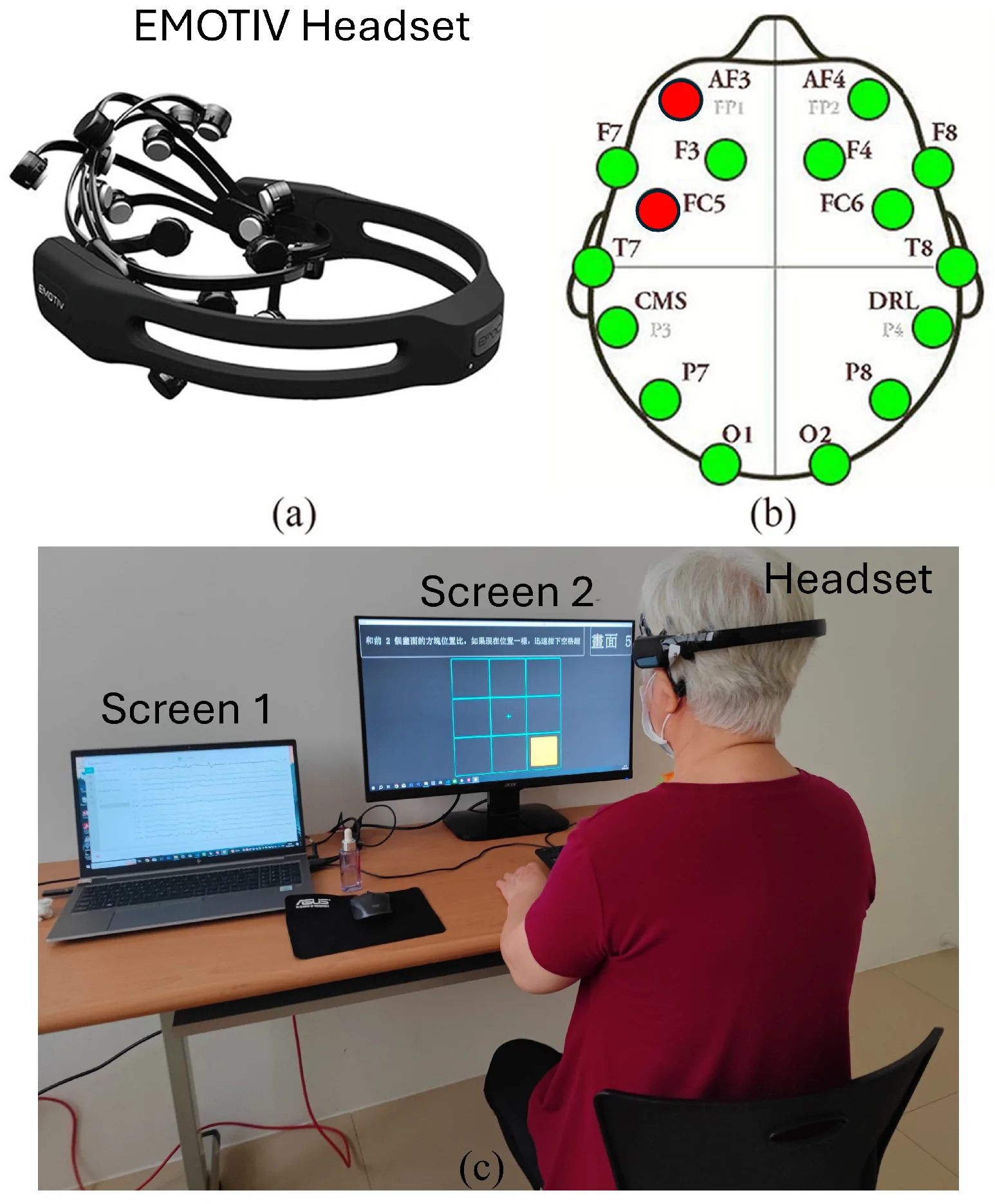
Apparatus and experimental setup. (**a**) EMOTIV EPOC X 14-channel headset. (**b**) 10–20 electrode montage with the NF electrodes highlighted in red (AF3, FC5). (**c**) Participant seated with the headset; Screen 1 (laptop) runs acquisition/processing, and Screen 2 presents the NF/N-back stimuli at ∼50 cm. The on-screen text visible on Screen 2 is in Traditional Chinese, the participants’ native language; it presents the N-back task instruction (press the spacebar when the current square position matches the position shown *N* screens earlier) and the trial counter.

EEG was recorded with a portable 14-channel EMOTIV EPOC X headset (EMOTIV Inc., San Francisco, CA, USA) (AF3, F7, F3, FC5, T7, P7, O1, O2, P8, T8, FC6, F4, F8, AF4; 128 Hz sampling). Electrode contact quality was monitored continuously in the EmotivPro software (version 3.2) and rebalanced (saline re-wetting/repositioning) whenever contact quality fell below 70%. Participants were seated upright in an adjustable chair at a desk. Experiments were conducted in a quiet room under controlled ambient lighting and temperature, with minimal environmental noise. Participants faced a stimulus display placed at ∼50 cm viewing distance. The experimental setup and layout are shown in Figure 2.

A laptop (Screen 1) ran the computer application and handled real-time acquisition, online processing, feedback rendering, and data logging; this display was used by the experimenter for monitoring. A separate participant-facing monitor (Screen 2), positioned ∼50 cm in front of the participant, presented both the NF game and the N-back stimuli. The user interface was rendered at 60 FPS. No physical responses were required during alpha-band NF. Responses during the N-back task were collected on a standard USB keyboard (target keypress with the dominant hand).

EEG streaming used the EMOTIV Cortex API. Event markers time-stamped in software (millisecond resolution) included the following: fixation onset, alpha-NF window onset, and each threshold-crossing events (car forwards/car backwards). For N-back: fixation onset, N-back stimulus onsets, and keyboard responses. N-back accuracy was recorded separately for each game.

### 2.4. Neurofeedback Task: Alpha-Inhibition Car Game

#### 2.4.1. Signal Feature and Target Sites

The NF loop targeted frontal alpha-band activity as a neurophysiological feature associated with top-down attentional control and inhibitory gating. Absolute alpha power (8–12 Hz) was continuously computed in real time, and the control signal was defined as the mean alpha power across AF3 and FC5. These electrodes overlie the frontal and left frontocentral cortex and have been consistently associated with attentional suppression and executive control processes.

Real-time alpha power was visualised on a side bar to provide continuous feedback on the alpha-band neurofeedback feature. Unlike previous studies that rely on a fixed reference such as resting-state peak alpha [51], we implemented a dynamic threshold using an adaptive Schmitt-trigger approach that adapted to each participant’s moment-to-moment baseline. This approach accounted for (i) intra-individual fluctuations, (ii) maintained task difficulty within an optimal challenge zone, and (iii) ensured that feedback remained sensitive and responsive across the session.

#### 2.4.2. Real-Time Estimator and Dynamic Thresholds

We used an adaptive Schmitt trigger to convert continuous alpha power into stable control signals. Unlike a single moving cutoff, a Schmitt trigger maintains two dynamic thresholds around a short-term baseline, producing a hysteresis band. This band prevents rapid on–off toggling (chatter) by requiring a stronger excursion to switch states than to remain in the current state.

EEG was buffered in 1s windows (128 samples) with a 1-sample slide (≈7.8ms).

Let ${\bar{A}}_{t}$ be the alpha mean from AF3 or FC5 at time *t*. The system maintained a FIFO list $L_{t}$ of the most recent 50 values $\left\{{\bar{A}}_{t-49},\ldots ,{\bar{A}}_{t}\right\}$. Define current attention, $C_{t}$, at time *t* as follows:

(1) $$C_{t}=\frac{1}{50}\sum_{k=0}^{49}{\bar{A}}_{t-k}.$$

#### Dynamic Thresholds (Hysteresis Band)

Upper and lower decision thresholds are set *around* the baseline $C_{t}$ (Equation (1)) to form a multiplicative hysteresis band (Equation (2)):

(2) $$T_{t}^{\mathrm{upper}}=\left(1+\kappa_{↑}\right)\;C_{t},\;T_{t}^{\mathrm{lower}}=\left(1-\kappa_{↓}\right)\;C_{t},$$

where $\kappa_{↑}=0.60$, and $\kappa_{↓}=0.40$ in our implementation. The instantaneous control $u_{t}$ follows the adaptive Schmitt policy:

(3) $$u_{t}=\left\{\begin{array}{cc} \mathrm{forward}, & {\bar{A}}_{t}>T_{t}^{\mathrm{upper}},\\ \mathrm{backward}, & {\bar{A}}_{t}<T_{t}^{\mathrm{lower}},\\ \mathrm{hold}, & \mathrm{otherwise}. \end{array}\right.$$

This creates a hysteresis band of width $H_{t}=T_{t}^{\mathrm{upper}}-T_{t}^{\mathrm{lower}}=\left(\kappa_{↑}+\kappa_{↓}\right)\;C_{t}$, adapting continuously to the participant’s moment-to-moment baseline and thereby stabilising the feedback while keeping the task in an optimal challenge zone.

Commands were sent to the UI during 10 s feedback epochs. The list updated by dropping the oldest 5 and appending 5 new values; end-to-end command latency was on the order of tens of milliseconds, dominated by window slide and frame refresh.

#### 2.4.3. Blocks, Cues, and Feedback

**Figure 3:**
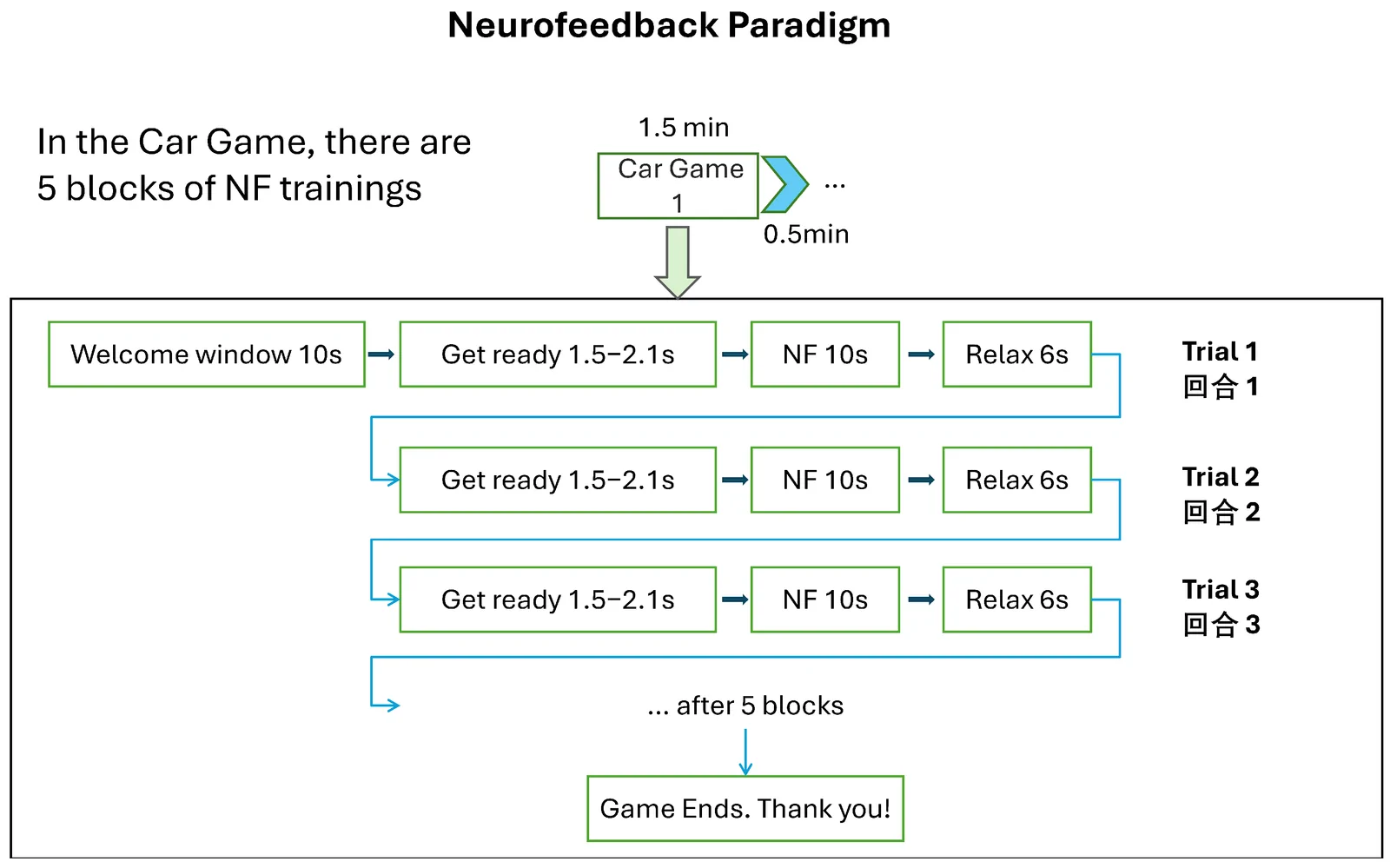
NF training paradigm timeline diagram showing five consecutive NF trials, each comprising preparation, NF training, and relaxation stages. The Chinese text below “Trial 1”–“Trial 3” is the Traditional Chinese translation of “Trial”, as shown to participants. The ellipses (…) indicate that the same trial cycle repeats until five blocks are completed.

**Figure 4:**
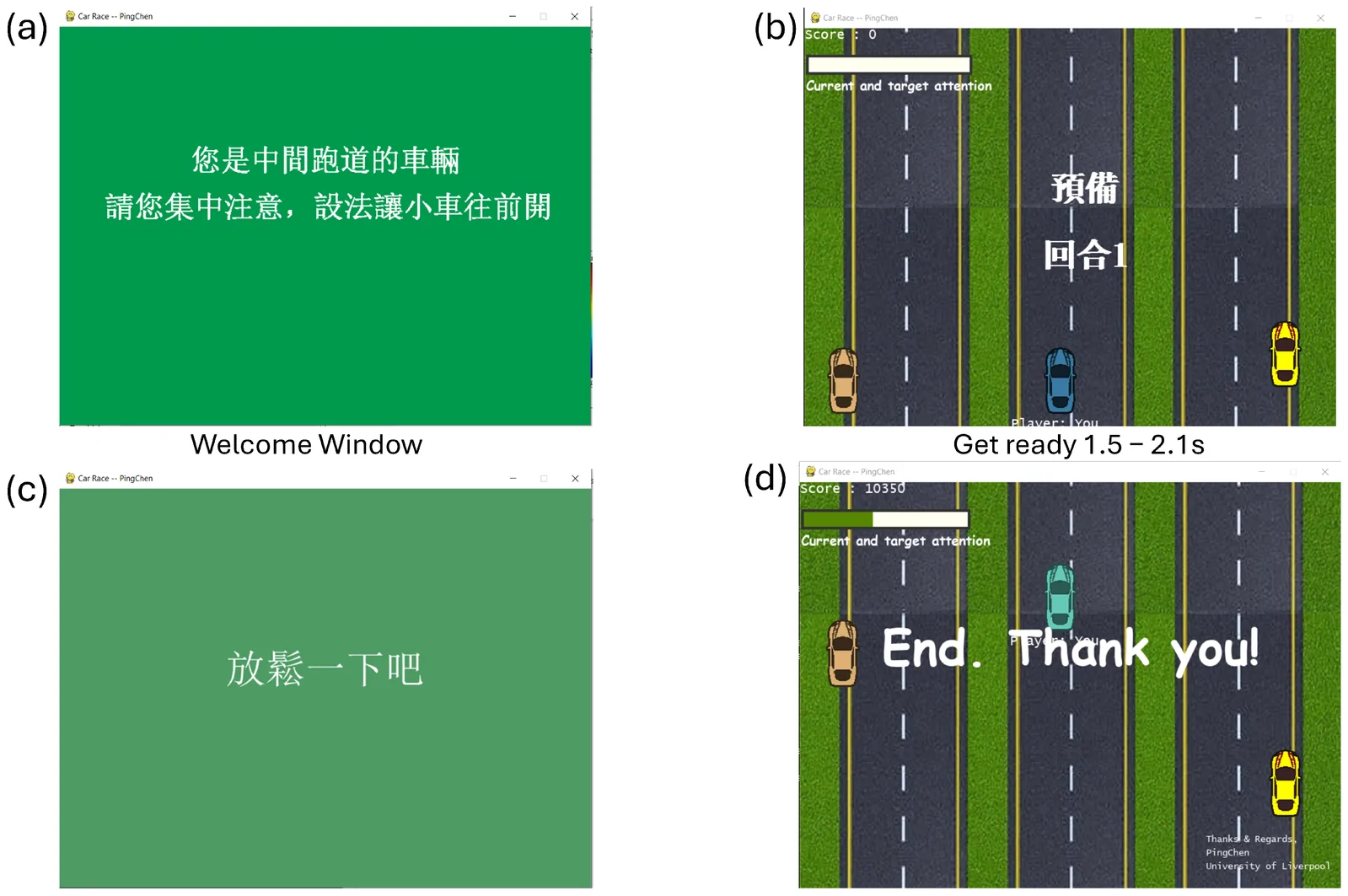
Screenshots of instruction windows throughout the NF protocol, including (**a**) welcome, (**b**) trial start, (**c**) relaxation, and (**d**) end messages. The on-screen text is in Traditional Chinese, the participants’ native language: the welcome window (**a**) instructs the participant to concentrate and try to drive the middle-lane car forwards, the trial-start window (**b**) reads “Get ready, Trial 1”, and the relaxation window (**c**) reads “Take a short rest”.

Each NF session consisted of short blocks with a brief preparation cue (jittered 1.5–2.1 s), a feedback epoch (10 s), and a rest screen (6–7 s). During feedback, control followed the adaptive Schmitt trigger policy defined in the previous section: the turquoise car moved forward (turquoise) when ${\bar{A}}_{t}>T_{t}^{\mathrm{upper}}=\left(1+\kappa_{↑}\right)C_{t}$, *backward* (pink) when ${\bar{A}}_{t}<T_{t}^{\mathrm{lower}}=\left(1-\kappa_{↓}\right)C_{t}$, and remained neutral within the hysteresis band $\left[T_{t}^{\mathrm{lower}},\;T_{t}^{\mathrm{upper}}\right]$. A minimum dwell time (200 ms) was enforced before allowing direction reversals to suppress chatter. Game start/end and car-direction events were timestamped for analysis. The five consecutive trials (prepare → feedback → rest) follow the timing structure in Figure 3, with the corresponding instruction windows in Figure 4.

### 2.5. Working Memory Task: Adaptive Spatial N-Back

#### 2.5.1. Stimuli and Timing

A 3 × 3 grid displayed a yellow square for 1750 ms followed by 1250 ms blank. Participants pressed the spacebar when the current position matched that from *N* trials earlier. Each N-back run began with a 9 s fixation.

#### 2.5.2. Adaptive Difficulty

The random repetition rate of the target squares is 30.5%. This parameter was established during the first batch recruitment N-back demonstration phase based on pilot observations. Each session comprised three training blocks; within each block, three *N*-levels (N≥1) were attempted for 1.5 min per level. At the end of each 1.5 min interval, the difficulty level *N* was adjusted according to the achieved accuracy (ACC) using the following criteria in Equation (4):

(4) $$N_{t+1}=\left\{\begin{array}{cc} N_{t}+1, & \mathrm{if}\;\mathrm{ACC}\geq 80\%\\ N_{t}-1, & \mathrm{if}\;\mathrm{ACC}<50\%\\ N_{t}, & \mathrm{if}\;50\%\leq \mathrm{ACC}<80\% \end{array}\right.$$

where $N_{t}\geq 1$. In practice, accuracy ≥80% advanced the task by one level. Accuracy below 50% was tolerated once, but if it occurred again in the following attempt, the level was reduced by one, with the constraint that *N* could never fall below 1. If accuracy fell between 50% and 80%, the current level was maintained. This mechanism allowed difficulty to increase without bound across sessions while preventing regression below the baseline level.

#### Manual Mode

A manual mode was available in which the adaptive adjustment was suspended and the *N*-level remained fixed regardless of performance. This mode was used only for demonstration purposes when introducing the task to participants and was not active during data collection.

#### Feedback and Logging

During the N-back task, participants received trial-wise visual feedback, with subtle colour cues indicating correct, incorrect, or missed responses. Feedback was brief and cleared before the onset of the next stimulus. At the end of each level, performance data were recorded, including the current *N* level, the sequence of level changes, and summary accuracy measures (number of hits, false alarms, misses, and overall accuracy). This ensured a complete record of both behavioural performance and the progression of task difficulty across the sessions.

### 2.6. EEG Recordings

We recorded the raw EEG signals using headset EMOTIV EPOC X. Raw EEG streams were acquired continuously and mapped to the python game modules. Online EEG for baseline, NF, and N-back were logged with synchronised event markers (fixation, stimulus onsets, and responses; car forward/backward; block boundaries). The UI operated at 60 Hz to minimise perceptual delay.

### 2.7. Outcome Measures

Consistent with the aims of a feasibility study, the outcomes are ordered as follows.

#### 2.7.1. Primary Outcome: Feasibility

Feasibility was defined before analysis as adherence to the 24-session schedule, operationalised as the proportion of planned sessions completed, and supported by tolerability (adverse events; session terminations) and successful data acquisition (proportion of sessions yielding usable EEG). This is the only outcome the present design is adequate to evaluate. No formal progression threshold was prospectively specified; feasibility was characterised descriptively using adherence, tolerability, and usable-data rates.

Adverse events were ascertained actively rather than by spontaneous report: at the end of every session, each participant was asked whether they had experienced any discomfort during that session, and any report was recorded. Enquiry was open-ended and was not structured by a symptom checklist, so the reported frequencies reflect symptoms participants were willing to describe in response to a general question.

#### 2.7.2. Secondary Outcomes (Exploratory): Oscillatory Measures

(i) Modulation of alpha-band power during the NF attentional-inhibition task, indexing the learned ability to regulate alpha; (ii) modulation of theta-band power during the N-back task, indexing working memory engagement; and (iii) N-back accuracy by difficulty level.

#### 2.7.3. Secondary Outcomes (Exploratory): Functional and Cognitive Measures

(i) Balance and gait stability, assessed with the Berg Balance Scale short form (BBS-3P) [52] and the 4-item Dynamic Gait Index (DGI-4) [53]. The BBS-3P was developed and validated in people with stroke [52], and its measurement properties may differ in healthy older adults, which should be borne in mind when interpreting the ceiling effects reported below; and (ii) cognitive performance, assessed with the Colour Trail Making Test parts A and B (CTMT-A/B) [54], measuring processing speed and visual attention (CTMT-A) and cognitive flexibility and working memory updating (CTMT-B).

All balance and gait assessments were administered by the same examiner at all three time points, removing between-examiner variability as a source of change. The CTMT was administered in the same form on all three occasions; alternate or parallel forms were not used. Repeated exposure to an identical test form is expected to produce practice effects, and the cognitive trajectories reported in Section 3.6 should be read with that in mind.

All secondary outcomes are treated as exploratory. In the absence of a control group, change in any of them is compatible with placebo, practice, or familiarisation effects as well as with the intervention, and no secondary outcome was designated as a basis for inference about efficacy.

### 2.8. Offline EEG Analysis

All electroencephalography (EEG) recordings were processed offline using the EEGLAB toolbox (version 2023.0) in MATLAB R2023a (The MathWorks, Inc., Natick, MA, USA). The pre-processing pipeline was designed to isolate clean, task-related oscillatory dynamics at the independent component (IC) level. Continuous raw EEG data were first re-referenced to the common average, band-pass filtered between 1 and 50 Hz using a zero-phase finite impulse response (FIR) filter, and notch filtered at 60 Hz to remove line noise. Prior to performing independent component analysis (ICA) [55], the continuous data were high-pass filtered at 1 Hz. The resulting ICs were classified using ICLabel, and components identified as non-brain sources (e.g., ocular, muscular, or cardiac artefacts) were rejected. All retained ICs were visually inspected to confirm appropriate spatial topography and spectral characteristics. These clean IC activations formed the basis for all subsequent analyses.

For feature extraction, the processed data were first segmented into task-specific epochs. In the NF task, analysis focused on two distinct regulation states indexed by specific event markers. This coding is used consistently throughout this manuscript:

- Event 6 denoted successful regulation: alpha power exceeded the adaptive upper threshold ($T^{upper}$), the car moved forward, and alpha was synchronised (increased).
- Event 7 marked unsuccessful regulation: alpha power fell below the lower threshold ($T^{lower}$), the car moved backward, and alpha was desynchronised (decreased).

Epochs were extracted from −0.3 to +1 s relative to the onset of these events. For the N-back task, epochs were extracted from −0.5 to +2 s relative to stimulus onset. Time–frequency decomposition was then conducted on these epochs using complex Morlet wavelets to compute event-related spectral perturbations (ERSPs). Absolute power values were calculated on a trial-by-trial basis and transformed to a decibel (dB) scale. A task-specific −200 ms baseline correction was applied using the pre-event interval. For both tasks, components were classified into eight regions of interest (ROIs) based on their scalp topographies: Left-Frontal, Right-Frontal, Left-Temporal, Right-Temporal, Left-Occipital, Right-Occipital, Left-Parietal, and Right-Parietal. Noisy and missing data were handled at three levels, and no imputation was performed at any of them. During recording, electrode contact quality was monitored continuously and remediated, and a session was repeated if adequate contact could not be restored. During pre-processing, components classified as non-brain sources were rejected, and the remainder were inspected visually; trials in which the recorded car movement contradicted the event being analysed were excluded. At the analysis stage, all tests used complete cases, so a participant contributed to a given region only if a usable component was recovered there; this is why the sample size differs between regions and is reported with each result.

Analyses were conducted at this regional level rather than as interpolated scalp maps. With 14 electrodes and wide inter-electrode spacing, a topographic map would imply a spatial resolution that the montage does not provide, and component-level data were not recovered at every region in every participant; the regional level is therefore the finest resolution that these data support. Data were also aggregated and summarised by training stages (Early: sessions 1–8; Middle: 9–16; Later: 17–24).

The analyses were divided according to the two task paradigms: (1) the NF task, focusing on alpha-band modulation (8–13 Hz) as a neurophysiological feature associated with attentional inhibition; and (2) the N-back task, targeting theta-band activity (4–7 Hz) as an indicator of working memory load and cognitive updating. Although different frequency bands were emphasised, both analyses were performed using a consistent pre-processing pipeline, identical baseline-correction windows, and stage-wise comparisons (Early, Middle, and Later). This ensured that the inference of task-specific frequency effects reflected functional distinctions rather than methodological bias. To further minimise bias, statistical thresholds, time–frequency decomposition parameters, and non-parametric comparisons were standardised across both tasks. Together, these procedures allowed a fair evaluation of how NF training and working memory engagement modulated oscillatory dynamics at both alpha and theta frequencies.

### 2.9. Feature Selection: Independent Components

ICA was employed to separate putative neural activity from common artefactual sources and to facilitate component-level analysis prior to oscillatory feature extraction. Each dataset was decomposed into statistically independent components (ICs), and the resulting sources were automatically classified using IClabel to assist in the identification of non-neural artefacts such as ocular or muscular activity. All components labelled as brain sources were subsequently subjected to detailed visual inspection to confirm physiologically plausible topographies and event-related spectral responses.

The selection of components for further analysis was based on two principal criteria: (1) the presence of distinct event-related potential (ERP) deflections that were temporally aligned with task events, and (2) the exhibition of stable spectral patterns across trials, such as alpha desynchronisation or theta synchronisation. Components meeting both criteria were considered reliable neural sources for investigating training-related oscillatory modulation.

Selection was applied blind to training stage and to behavioural outcome. Of the 14 components available from each decomposition, 2 to 4 were retained per participant per session, 4 where the raw recording was clean and two where signal quality was poorer; across the eight sessions comprising a training stage, this yielded approximately 25–30 retained components per participant per stage. Because all components contributing to a given region and stage are averaged into a single value per participant before any test, the number retained affects the precision of that average rather than the degrees of freedom of any reported statistic.

**Figure 5:**
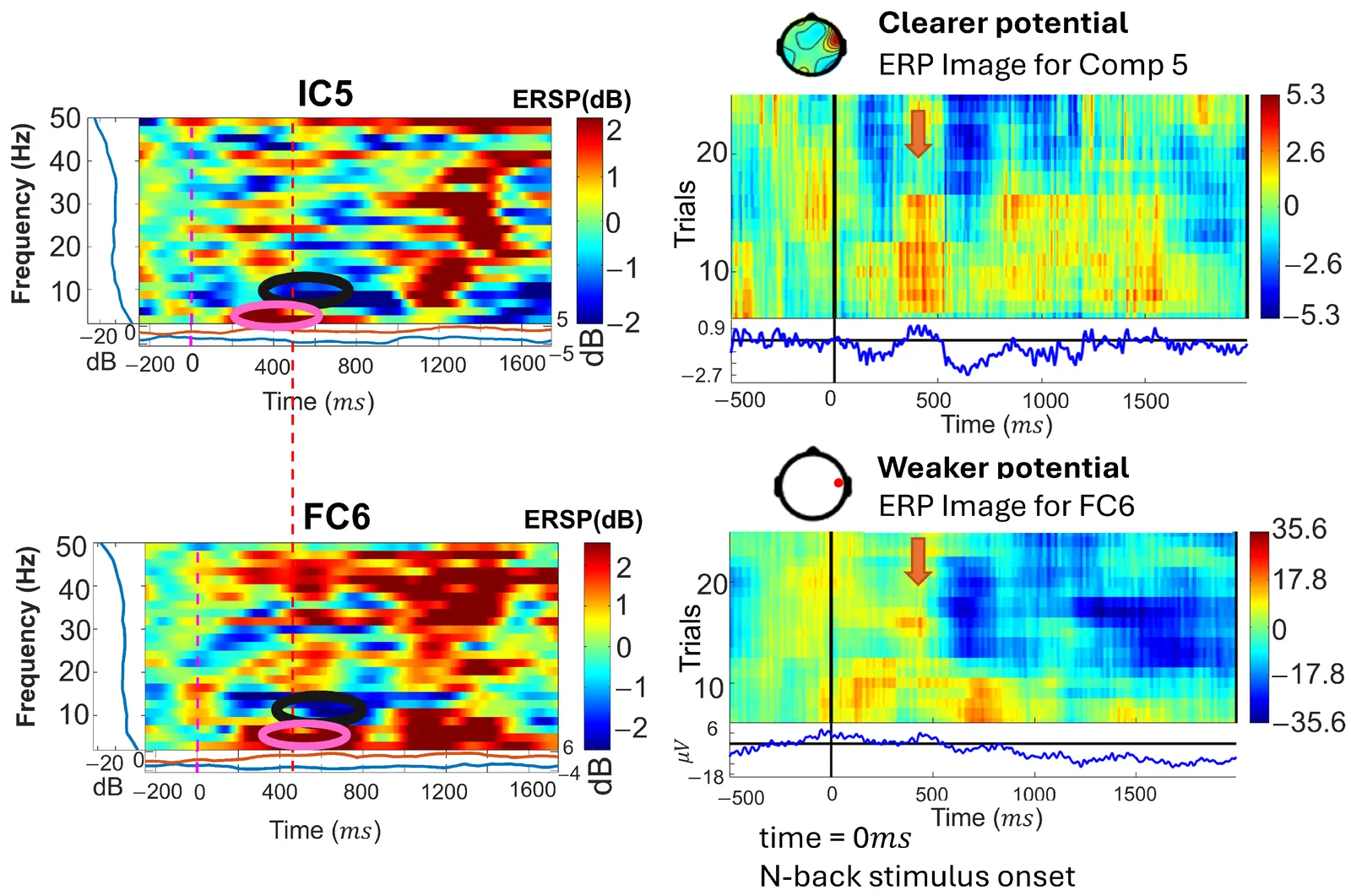
Comparison between ICA-derived and raw channel EEG for one participant (Subject 104). In the left panels, the magenta dashed line marks stimulus onset (0 ms), the red dashed line marks the component’s P3 peak latency, and the pink and black ellipses mark theta-band enhancement and alpha-band suppression, respectively. In the right panels, the vertical black line marks stimulus onset, and the orange arrows indicate the post-stimulus ERP deflection. The independent component (Comp. 5, peaking near FC6) shows clearer trial-level ERP structure and better frequency resolution than the corresponding channel signal. This panel is illustrative of the pre-processing pipeline only; no inference is drawn from it, and all statistical results reported in this manuscript are computed across participants.

Figure 5 illustrates a typical comparison between a component-level signal (Component 5, localised near FC6 as shown in the topography) and its corresponding raw channel data from a subject during cognitive training. The ICA-derived representation demonstrates markedly enhanced signal clarity, with trial-wise ERP structure and well-defined frequency dynamics that are largely obscured at the channel level. Specifically, the component exhibits clear theta-band enhancement (pink ellipse) and alpha-band suppression (black ellipse) following stimulus onset, while the raw channel trace shows blurred and noisy temporal dynamics. This comparison highlights the advantage of performing oscillatory analyses at the component rather than channel level, improving separation of putative neural and artefactual components while retaining the limited spatial interpretability of the 14-channel montage.

The refined component-level approach therefore provided a robust foundation for the subsequent analyses of NF and N-back training tasks. By isolating components that exhibit distinct oscillatory signatures, this pre-processing supported the interpretation that observed changes in alpha and theta power reflect putative neural activity rather than artefactual fluctuations at the scalp level.

#### 2.9.1. Attention Feature

We hypothesised that participants could learn to deliberately self-regulate their alpha-band (8–13 Hz) activity and that the success of this modulation would correlate with behavioural measures of attention, balance, and gait. To test this, analysis of the NF task targeted the modulation of the alpha band (8–13 Hz) during deliberate self-regulation. Trials were time-locked to two distinct events: event 6, indexing alpha synchronisation, and event 7, indexing alpha desynchronisation. To accommodate inter-session variability in neural transmission and sensorimotor delays, the post-event analysis window was defined for each IC based on expert annotation of its response latency (typically ≥150 ms). Within each ROI, mean alpha power was computed at the component level, averaged across trials, and then aggregated by the predefined training stages. Alpha synchronisation was operationalised as an increase in alpha power following event 6, and desynchronisation was operationalised as a decrease following event 7. To prevent effect cancellation, trials in which the recorded car movement contradicted the event being analysed were excluded. These stage-wise modulation patterns were then compared non-parametrically and related to behavioural measures of attention, balance, and gait.

#### 2.9.2. Working Memory Feature

To test the hypothesis that working memory load-related theta dynamics are specifically linked to P3-generating neural sources, we performed a targeted feature extraction. For the N-back task, feature extraction targeted theta (4–7 Hz) dynamics associated with working memory load. Before performing time–frequency decomposition, each independent component (IC) ERP was first inspected visually to identify task-related activity in the 250–400 ms range. Components showing clear P3-like deflections were selected for further analysis.

This component-based selection strategy is highly effective for noise exclusion. It allows for the isolation of task-relevant neural signals while segregating artefacts into separate components, thus avoiding the need to exclude entire noisy epochs. This preservation of data is a critical advantage, particularly when working with a limited number of recordings.

In cases where polarity was ambiguous, the latency of the maximal absolute amplitude within this range was used. This approach ensures that the subsequent time–frequency analysis is aligned to the functionally relevant ERP feature rather than a fixed latency across subjects. The polarity of an IC ERP can invert depending on the dipole orientation of its cortical generator. Therefore, analysing the magnitude (absolute amplitude) is a standard practice [56,57]. This procedure interprets positive and negative deflections as the same phase-locked neural process. This approach aligns with previous findings that a single neural source can exhibit polarity differences across different cortical regions [29]. This hypothesis-driven selection was designed to link P3 processes with theta dynamics, intentionally excluding components that did not meet this criterion.

Stimulus-locked ERSPs were then computed from −0.5 to +2 s. To account for inter-individual and inter-session variability in processing speed, the analysis window was defined as a 400 ms interval centred on each IC’s P3 peak latency (latency ±200 ms). Within each ROI, mean theta power (dB) was extracted from the defined window, averaged per participant and training stage, and subsequently related to N-back accuracy across difficulty levels. Correlations with executive-function indices (Trail Making Test A/B) were further examined to assess the functional relevance of this oscillatory modulation.

This component-level, event-aligned time–frequency approach yielded spatially specific, temporally precise, and behaviourally interpretable neural features to evaluate longitudinal changes and examine the mechanisms underlying cognitive-motor improvements.

### 2.10. Statistical Analysis

All analyses were conducted in MATLAB R2023a using the Statistics and Machine Learning Toolbox with custom scripts and EEGLAB for EEG processing. Several measures were not normally distributed, so non-parametric methods were used throughout.

#### 2.10.1. Unit of Analysis

Every inferential test reported in this manuscript is at the participant level. EEG features were reduced to one value per participant, region, and stage before entering any test; single-trial data were used only to compute those values, never as independent observations. Where a figure shows data from one participant, it is labelled as illustrative, and no inference is drawn from it. The sample size for each test is stated with the result and differs between regions because a usable component was not recovered at every site in every participant.

#### 2.10.2. Stage Effects

Within-participant change across the three predefined stages (Early: sessions 1–8; Middle: 9–16; Later: 17–24) was assessed with Friedman tests, followed by Wilcoxon signed-rank tests for the three pairwise contrasts (Early vs. Middle, Middle vs. Later, and Early vs. Later). Effect sizes are matched-pairs rank-biserial correlations, reported with 95% confidence intervals obtained by bias-corrected and accelerated (BCa) bootstrap with 20,000 resamples in which participants, not observations, were resampled. For the regional EEG contrasts, Benjamini–Hochberg false discovery rate correction was applied across all 24 region × contrast cells (the eight region–band combinations with usable components, each tested at three stage contrasts) and the outcome is reported per cell.

#### 2.10.3. EEG–Behaviour Associations

These were computed two ways, and both are reported. The stage-pooled Spearman correlation treats each participant’s three stage observations as independent; it is the analysis used in the original submission and is retained only for comparison, since it inflates the effective sample size from 20 participants to 60 observations. The repeated-measures correlation (rmcorr) fits a common slope with a participant-specific intercept (df=39) and respects the repeated-measures structure; inference rests on this estimate. A Spearman partial rank correlation on Early-to-Later change scores was additionally used to test whether alpha modulation was associated with balance change independently of working memory gains.

#### 2.10.4. Multiplicity

No correction was applied to the family of 16 band × region × outcome correlations (two bands × two frontal regions × four behavioural outcomes). Including the behavioural and regional EEG analyses, 88 tests are reported in total. All tests were two-sided. *p*-values are therefore descriptive rather than inferential, and interpretation rests on the effect sizes and their confidence intervals; *p*-values below 0.001 are reported as p<0.001.

#### 2.10.5. Pre-Specification

The outcome hierarchy, the three training stages, the regions of interest, the stage contrasts, and the non-parametric tests used to evaluate them were all defined before the data were analysed. The emphasis on right-frontal alpha is the exception: it arose from the data rather than from the training target and is identified as exploratory wherever it appears.

#### 2.10.6. Sample Size and Blinding

No a priori sample size or power calculation was performed. The sample was determined by feasibility considerations rather than by a target effect size, which is appropriate for a study whose purpose is to establish deliverability and to estimate parameters for a subsequent trial; we report effect sizes with confidence intervals in place of hypothesis-test power. Post hoc power was not computed, as it is a deterministic function of the observed *p*-value and adds no information beyond the intervals reported here. Behavioural outcome assessment was not blinded at the point of administration, since the same team delivered the intervention. Statistical analysis was conducted blind to participant identity and to stage–outcome pairing. The absence of assessor blinding at administration is acknowledged as a source of potential detection bias.

## 3. Results

### 3.1. Participant Characteristics

Baseline characteristics of the enrolled cohort are summarised in Table 1. Participants were healthy, cognitively intact, and not selected for risk of fall. Both abbreviated functional instruments showed restricted baseline range: before training began, 2 of 20 participants (10%) already scored at the ceiling of the BBS-3P (28 of 28), and 6 of 20 (30%) scored at the ceiling of the DGI-4 (12 of 12), so the scope for measurable improvement on these scales was limited by design. This constraint should be borne in mind when interpreting all subsequent balance and gait findings.

### 3.2. Feasibility

Feasibility outcomes indicated that the system could be implemented reliably in this cohort. The BCI platform delivered real-time neurofeedback with an end-to-end latency of less than 250 ms, supporting near-immediate coupling between neural activity and visual feedback. Most participants reported that the game interface was straightforward to use, with 88% indicating that they could readily control the game object and respond to the visual feedback.

Recruitment and screening were feasible: 22 individuals were screened, and 2 were excluded due to pre-existing neurological conditions (dementia and poliomyelitis), resulting in 20 participants enrolled. Adherence to the training schedule was high, with 100% of planned sessions completed for both the neurofeedback and N-back components and no deviations requiring session termination. The delivered training dose was consistent, comprising a mean of five neurofeedback car-racing games and nine N-back games per session. Mean time-on-task was 10.2 min per neurofeedback session and 12.4 min per N-back session.

The protocol was well tolerated. No participants reported headache or discomfort related to the headset, and no sessions were cancelled. In terms of user feedback, 70% of participants gained confidence through repeated practice, and 75% reported high engagement with the game interface. Visual fatigue was reported by approximately 55% of participants, revealing sustained visual attention demands during training.

### 3.3. EEG Analysis in the Neurofeedback Task

The NF experiment investigated longitudinal modulation of cortical alpha dynamics during attention-based self-regulation. EEG data were first decomposed using ICA to separate neural and non-neural sources. Components were automatically labelled with IClabel and subsequently verified through visual inspection of ERPs and spectral profiles.

Both event markers are used in this analysis, at different points, and the distinction matters for interpreting what follows. Component selection used event 7: an independent component was retained only if it showed a clear event-locked deflection following the backward-movement marker, which identifies components genuinely responsive to the feedback contingency. The alpha modulation reported below was then quantified at event 6, the successful-regulation marker. Selection was performed blind to training stage and to behavioural outcome, so that although the absolute alpha values are conditioned on the selection step, the stage contrasts and the EEG–behaviour associations are not.

Single-trial alpha power (8–13 Hz) was computed using complex Morlet wavelets and expressed in decibels (dB) relative to a baseline window (−200 ms to 0 ms) and then averaged within participant, region, and training stage (Early, sessions 1–8; Middle, sessions 9–16; Later, sessions 17–24).

**Figure 6:**
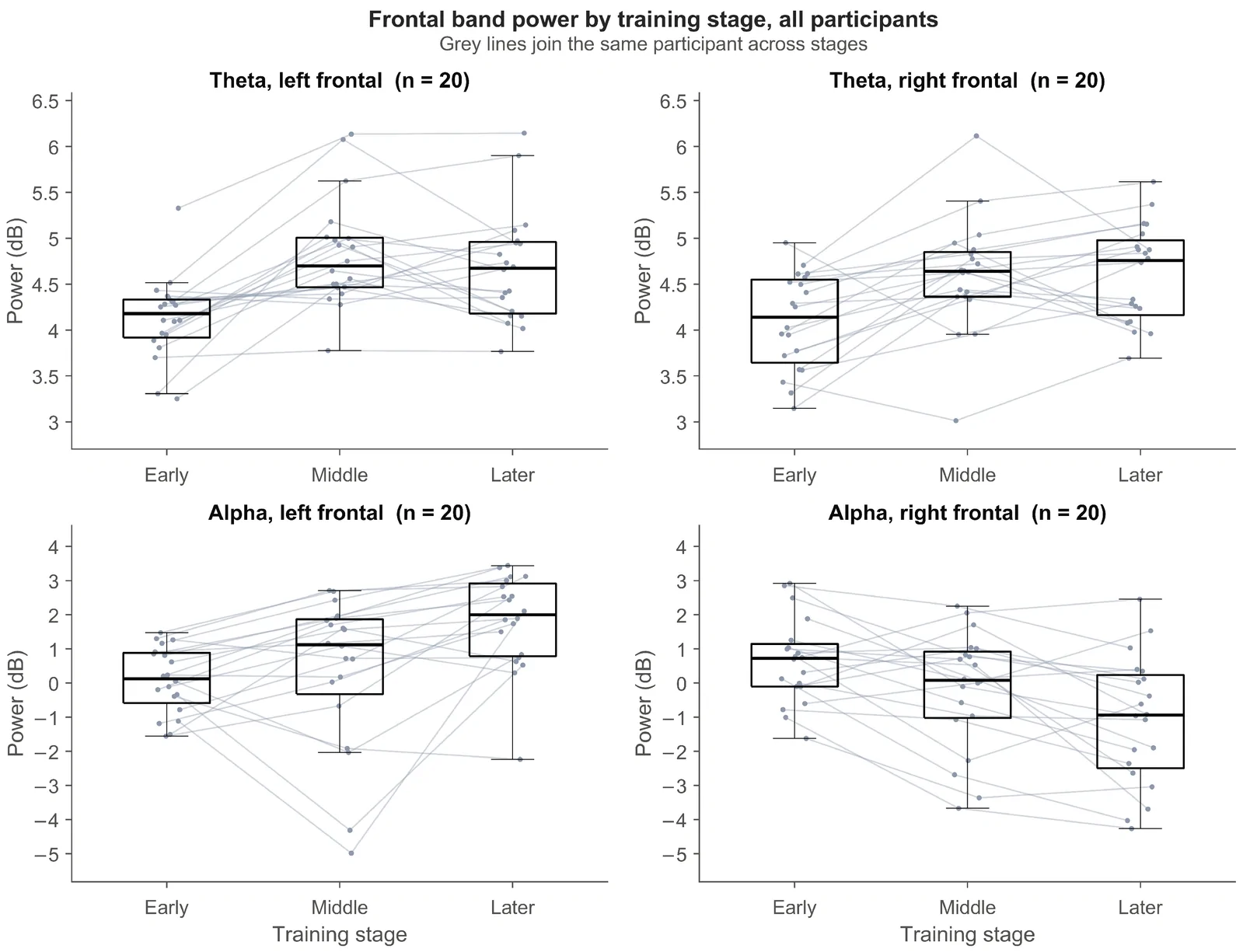
Frontal alpha and theta power at each training stage (n=20). Stages are defined by session number: Early, sessions 1–8; Middle, sessions 9–16; Later, sessions 17–24. Grey lines join the same participant across stages; boxes show the median and interquartile range. The two hemispheres share a y-axis within each band. Theta results are described in Section 3.4.

Alpha power during the successful regulation phase (Event 6) was analysed across the Early, Middle, and Later training stages at the participant level (n=20). Figure 6 shows the distribution at each stage for both bands and both frontal regions, with one line per participant.

**Figure 7:**
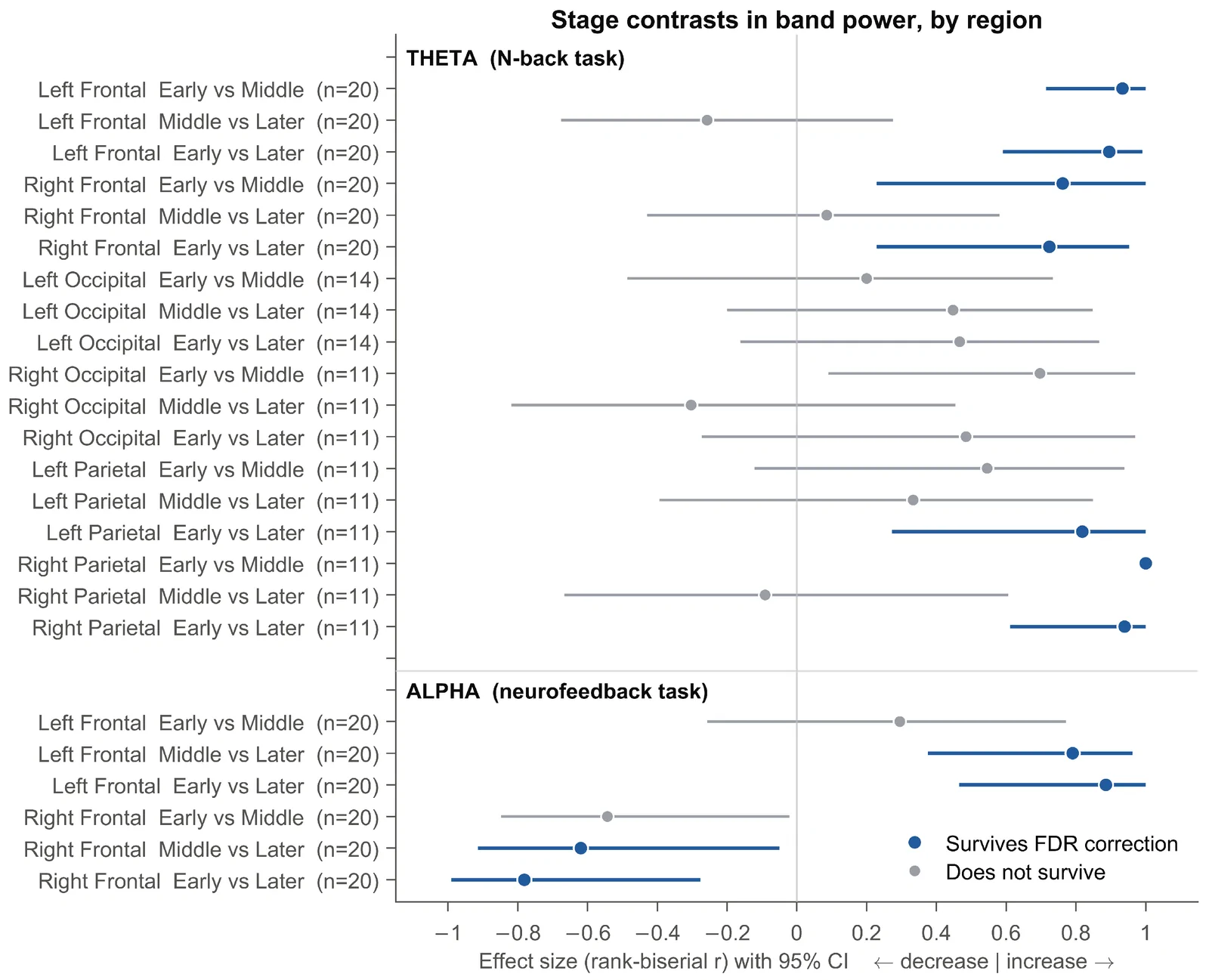
Stage contrasts in band power by region. Points are matched-pairs rank-biserial effect sizes with 95% BCa confidence intervals; positive values indicate an increase in power. Colour marks whether a contrast survives Benjamini–Hochberg correction across all 24 contrasts shown. *n* differs between regions because not every participant yielded a usable component at every site.

The two hemispheres moved in opposite directions. Left-frontal alpha, the region targeted by the neurofeedback contingency, rose steadily across training (median of 0.13, 1.12, and 2.00 dB at Early, Middle, and Later). The Early-to-Middle step was small and its interval included zero, but the Middle-to-Later and Early-to-Later increases were substantial and survived correction for multiple comparisons (Figure 7). The direction of this change matches the training target.

Right-frontal alpha fell over the same period (median 0.72, 0.08, and −0.94 dB), with the Middle-to-Later and Early-to-Later decreases surviving correction. Because the neurofeedback contingency reinforced alpha increase at a left-frontal site only, this contralateral decrease was neither trained nor predicted, and we treat it as an exploratory observation throughout.

Figure 7 summarises every regional contrast tested in both bands, with effect sizes, intervals, and the outcome of correction across all 24 comparisons. Regional coverage is uneven: the frontal regions retain all 20 participants, whereas the parietal and occipital regions retain 11–14, since a usable component was not recovered at every site in every participant. The parietal contrasts that survive correction therefore rest on roughly half the cohort and are not interpreted further. Regional labels denote the scalp sites at which component activity was strongest; no source localisation was attempted.

### 3.4. EEG Analysis in the N-Back Task

EEG data collected during N-back training were analysed to characterise oscillatory dynamics related to working memory. Theta-band (4–7 Hz) power was extracted from independent components (ICs) exhibiting clear P3-like activity within the left- and right-frontal ROIs, using the same ERP-informed ERSP method described previously for both hemispheres; this selected feature is referred to below as P3-associated frontal theta. The analysis window was defined as a 400 ms interval centred on each component’s peak latency (latency ±200 ms), and power values were expressed in decibels (dB) relative to the pre-stimulus baseline.

Theta power followed the same stage-dependent pattern in both hemispheres (Figure 6, upper row). In the left-frontal region the median rose from 4.18 dB at Early to 4.70 dB at Middle and was 4.67 dB at Later; in the right-frontal region, the corresponding values were 4.14, 4.64, and 4.76 dB. In both regions the Early-to-Middle and Early-to-Later increases were large and survived correction for multiple comparisons, whereas the Middle-to-Later change was negligible, and its interval spanned zero (Figure 7).

Unlike the original submission, which reported the left-frontal region only, both hemispheres are shown here. Their trajectories are closely matched, so these data give no indication that the theta increase was lateralised.

These results indicate an enhancement of frontal theta activity during the middle phase of training, which is consistent with increased working memory engagement as participants adapted to the N-back task and a plateau thereafter that is compatible with a transition from effortful processing to more efficient recruitment [47,58]. Both readings are interpretations of an uncontrolled time course, and neither can be distinguished from familiarisation with the task.

### 3.5. N-Back Level Performance Analysis

Behavioural performance across N-back difficulty levels (1 to 6) was evaluated using repeated-measures non-parametric tests. Significant stage effects were observed at lower loads:

- For 1-back: $\chi^{2}\left(2\right)=14.18$, p<0.001;
- For 2-back: $\chi^{2}\left(2\right)=13.24$, p=0.001;
- For 3-back: $\chi^{2}\left(2\right)=6.38$, p=0.041.

No significant effect was found for 4-back ($\chi^{2}\left(2\right)=0.20$, p=0.905). Statistical analysis was not possible for 5-back and 6-back due to limited records.

Post hoc Wilcoxon signed-rank tests revealed consistent accuracy gains across stages for the 1-back to 3-back levels. For 1-back, accuracy increased significantly from Early to Middle (p=0.032), Early to Later (p=0.001), and Middle to Later (p=0.023). For 2-back, improvements occurred from Early to Middle (p=0.004) and Early to Later (p<0.001). Similarly, 3-back accuracy improved from Early to Middle (p=0.017) and Early to Later (p=0.017), indicating rapid initial adaptation followed by performance stabilisation.

**Figure 8:**
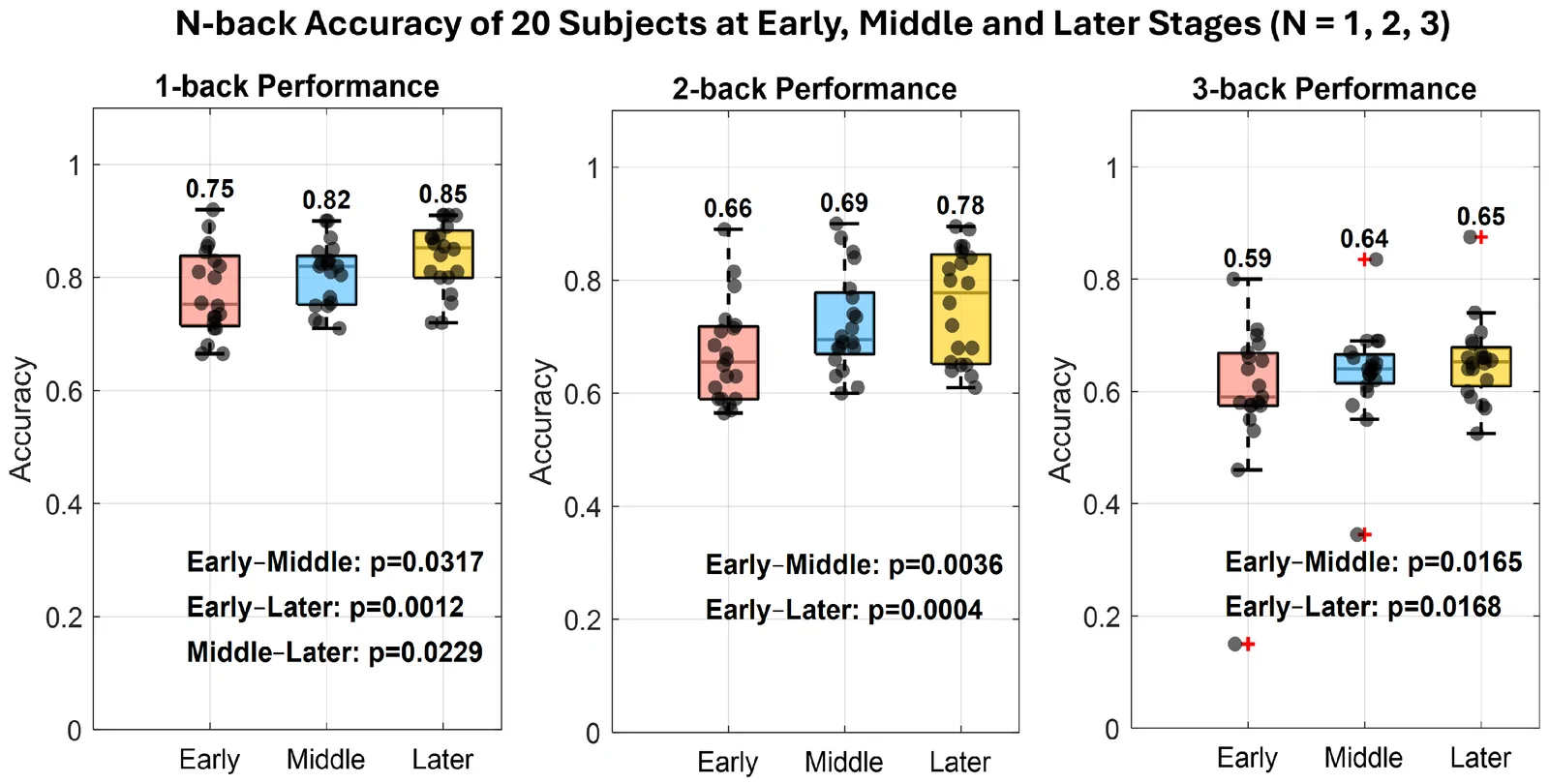
Box plots of task accuracy for 1-back, 2-back, and 3-back conditions across Early, Middle, and Later stages (n=20). Significant improvements across stages (p<0.05) are indicated. Grey dots show individual participants’ accuracies, and red plus signs mark outliers.

Figure 8 presents the accuracy distributions across stages for the loads all participants attempted. Performance was near ceiling at 1-back and 2-back and declined at higher loads. Accuracy at 4-back, 5-back, and 6-back is not plotted, because the number of participants reaching those loads falls away sharply (Table 2): fifteen reached 4-back, four completed 5-back, and one progressed to 6-back. No stage comparison was performed at 5-back or 6-back, and a box plot drawn from four participants, or from one, would invite an interpretation the data cannot support. For completeness, accuracy at 4-back did not improve across stages, and median accuracy at 5-back and 6-back is not reported for the same reason.

Reduced performance at higher N-back loads is qualitatively compatible with capacity-limited accounts such as the Compensation-Related Utilisation of Neural Circuits Hypothesis (CRUNCH) [49], which proposes that older adults increase neural recruitment to meet task demands up to a critical threshold, after which behavioural performance plateaus or deteriorates as cognitive resources are exhausted. However, the small and selectively surviving samples at 5-back (n=4) and 6-back (n=1) preclude meaningful evaluation of this hypothesis, which therefore remains contextual rather than tested here.

Table 2 summarises the distribution of the maximum achieved N-back levels, indexing individual working memory capacity within the cohort. All twenty participants were able to complete at least up to 3-back, whereas only a cognitively more resilient subset progressed to higher levels. The selective progression to 4-back and above therefore introduces survivorship bias, as participants who advanced further were those who could tolerate higher cognitive demands.

### 3.6. Behavioural Results

Behavioural performance was evaluated across three stages of the 24-session BCI training programme (Early: sessions 1–8; Middle: 9–16; Later: 17–24) using four validated assessments: the Berg Balance Scale short form (BBS-3P), the four-item Dynamic Gait Index (DGI-4), and the Colour Trail Making Tests A and B (CTMT-A/B). Wilcoxon signed-rank tests were performed for each adjacent stage pair (Early vs. Middle; Middle vs. Later) and between the earliest and latest stages (Early vs. Later).

Distributions at each stage are given in Table 3, and the corresponding stage contrasts are given in Table 4. Scores moved in the direction of improvement on all four measures, with the most robust changes emerging between the Middle and Later stages and being sustained through the end of training. For balance performance, BBS-3P improved from the Early to Middle stage (r=0.92, 95% CI [0.50, 1.00], p=0.002) and from the Early to Later stage (r=0.90, 95% CI [0.56, 1.00], p=0.001), whereas the Middle to Later change was smaller, and its interval included zero (r=0.59, 95% CI [−0.09, 0.92], p=0.078). These changes were, however, confined to a narrow range of the scale: the median rose from 26 to 28 of a possible 28, and the proportion of participants at ceiling rose from 10% to 65% (Table 3).

DGI-4 exhibited a later-emerging improvement profile. The Early-to-Middle interval included zero (r=0.67, 95% CI [0.00, 1.00], p=0.052), while the Middle-to-Later and Early-to-Later contrasts were larger (p=0.004 and p=0.001). The rank-biserial estimates for these two contrasts reach the boundary value of 1.00 because every participant whose score changed did so in the same direction; with only 10 and 13 of 20 participants showing any change at all and 75% at ceiling by the Later stage, these estimates should be read as indicating a consistent direction of change rather than a precisely estimated magnitude.

#### Clinical Interpretation of the Functional Changes

**Figure 9:**
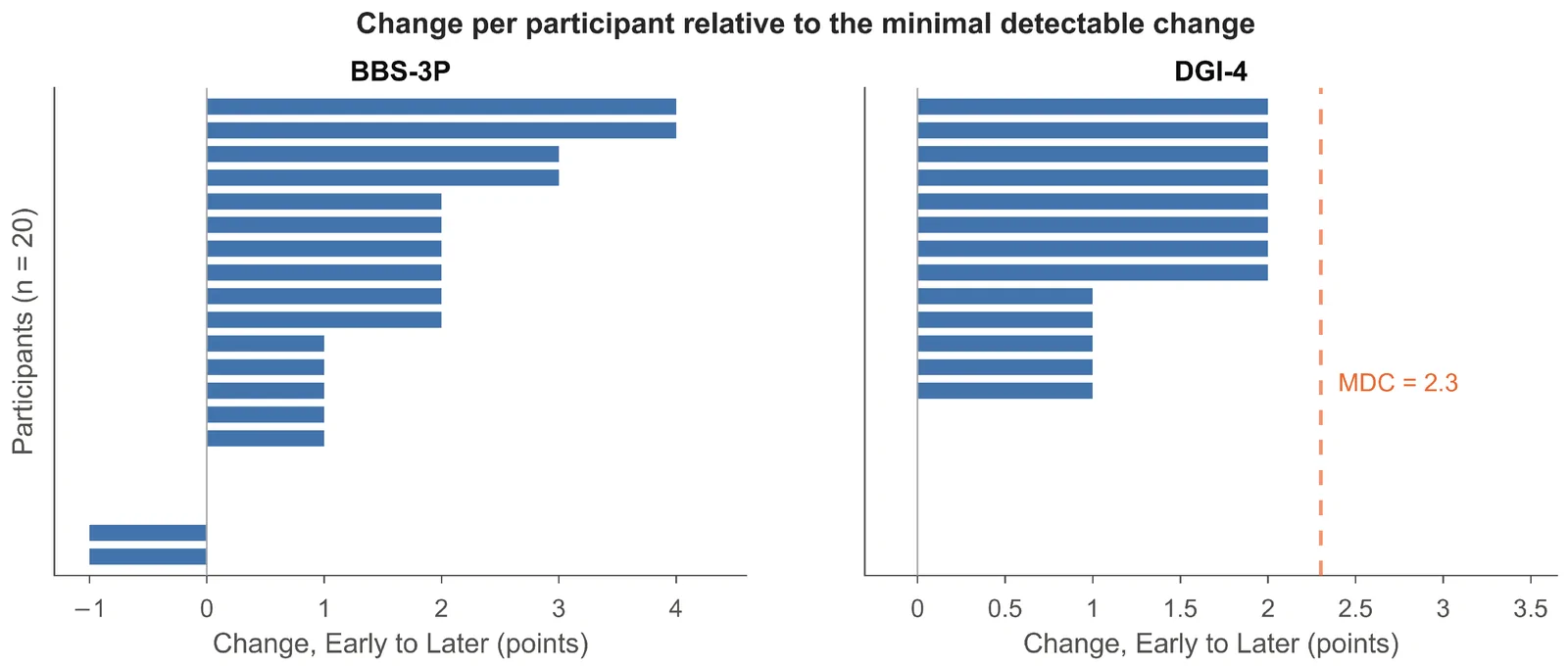
Change per participant from Early to Later on the two functional scales (n=20), sorted by magnitude. The dashed line marks the minimal detectable change in the DGI-4 (2.3 points [53]). No minimal detectable change has been published for the abbreviated Berg form in a comparable population, so none is drawn on that panel.

The minimal detectable change for the DGI-4 is 2.3 points [53]. In this cohort the median Early-to-Later change was 1 point and the largest was 2 points, so no participant reached that threshold (Figure 9). For the BBS-3P the median change was 1.5 points on a 28-point scale; no minimal detectable change has been published for the abbreviated form in a comparable population, and values derived from the full 56-point instrument in clinical samples do not transfer to a scale with a different item set and response range. The functional changes reported above should therefore be interpreted as nominal rather than clinically meaningful, consistent with a healthy cohort already close to the ceiling of both scales at baseline (Table 1).

Cognitive performance followed a more uniform pattern of improvement across training stages. Before these changes are described, it must be noted that the identical CTMT form was administered on all three occasions, and repeated exposure to the same form is expected to produce large gains in completion time independently of any change in underlying ability; intervention and practice effects therefore cannot be separated in what follows. Completion times for CTMT-A, indexing visual scanning, psychomotor speed, and sustained attention decreased across all pairwise comparisons (all p<0.001; all 20 participants improved at each contrast), and CTMT-B, which places greater demands on set-shifting, executive control, and working memory, improved likewise (Early vs. Middle and Middle vs. Later: p=0.003; Early vs. Later: p<0.001). Unlike the functional scales, neither CTMT-A/B measure was subject to a ceiling, and the effect-size intervals for CTMT-B are correspondingly wider and better estimated. The magnitude of the CTMT-A change is substantial: median completion time fell from 96.0 to 43.0 s, a reduction of more than half. This pattern is consistent with the working memory demands of the N-back task, but without a control group, the practice and intervention explanations cannot be distinguished, and the cognitive results should be interpreted accordingly.

Taken together, scores moved in the direction of improvement on all four behavioural measures over the training period. Given the single-arm design, the limited headroom on both functional scales, and the repetition of identical test forms, these changes describe the trajectory of the cohort during training; they cannot be attributed to the intervention, and the associations examined below are correspondingly exploratory.

### 3.7. Neuro–Behavioural Correlations

Associations between regional EEG power and the behavioural outcomes were examined in two ways, and both are reported in Table 5 and Table 6 so that they can be compared directly. The first is the stage-pooled Spearman correlation, which treats each participant’s three stage observations as independent. This is the analysis reported in the original submission; it inflates the effective sample size from 20 participants to 60 observations and violates independence, so it is retained only for comparison. The second is the repeated-measures correlation (rmcorr), which fits a common slope with a separate intercept per participant (df=39) and therefore respects the repeated-measures structure. Inference should rest on the rmcorr coefficients. Confidence intervals are 95% bias-corrected and accelerated (BCa) intervals obtained by resampling participants rather than observations. All correlations in this section are exploratory and hypothesis-generating.

#### 3.7.1. Alpha Power During Neurofeedback

Table 5 summarises stage-pooled correlations between frontal alpha power during neurofeedback and behavioural outcomes. Alpha values were expressed in dB relative to a pre-event baseline, such that more negative values reflect stronger task-related alpha desynchronisation. Alpha power was averaged across the Early, Middle, and Later stages, respectively, for correlation analysis.

The largest association was between right-frontal alpha and BBS-3P, where greater right-frontal desynchronisation co-occurred with better balance scores ($r_{\mathrm{rm}}=-0.64$, p<0.001). We emphasise that this relationship was not pre-specified: the neurofeedback contingency explicitly reinforced enhancement of alpha power at the left-frontal site, and the association reported here is in the contralateral hemisphere and in the opposite direction. It is therefore an unexpected exploratory observation rather than confirmation of the hypothesised mechanism, and it is reported as hypothesis-generating. Frontal alpha desynchronisation has elsewhere been interpreted as a marker of increased sensorimotor engagement during movement preparation and postural control in older adults [43], but the present design cannot test that interpretation, and a 14-channel montage cannot localise the underlying generator.

The associations were not confined to balance. Right-frontal alpha also tracked DGI-4 ($r_{\mathrm{rm}}=-0.48$, p=0.001) and CTMT-A ($r_{\mathrm{rm}}=+0.47$, p=0.002), and left-frontal alpha tracked DGI-4 ($r_{\mathrm{rm}}=+0.42$, p=0.006) and CTMT-A ($r_{\mathrm{rm}}=-0.41$, p=0.008). Because these coefficients share a sign structure across outcomes within each hemisphere, part of this pattern is likely to reflect general improvement over the training period rather than coupling that is specific to any single behavioural domain. Two features nonetheless stand out from the rest of the family. The right-frontal alpha–BBS-3P coefficient is the largest in the entire dataset and the only one for which the participant-level estimate substantially exceeds its stage-pooled counterpart, and right-frontal alpha–DGI-4 is the next largest. Both retain moderate-to-large magnitudes after the repeated-measures structure is accounted for. Eight alpha cells were examined without correction for multiplicity, so these are candidate features identified by screening rather than established associations; they are, however, the features this dataset nominates for prospective testing.

To investigate whether neurofeedback-specific alpha modulation predicts postural stability independently of general cognitive gains, we computed Spearman partial rank correlation. It is calculated on change scores (Later vs. Early) for each participant.

As shown in Table 7, the correlation between change in right-frontal alpha and change in BBS-3P was moderate but did not reach the nominal threshold (ρ=−0.40, p=0.170), while change in N-back accuracy showed a comparable association with BBS-3P (ρ=−0.52, p=0.067). Controlling for N-back accuracy change, the alpha–BBS-3P association attenuated further ($\rho_{\mathrm{partial}}=-0.32$, p=0.310).

This analysis and the repeated-measures correlation reported earlier (r=−0.64, Table 5) answer two different questions, and the difference between them explains why the two coefficients differ. The repeated-measures correlation asks whether a participant’s balance score is higher at the stages where their own right-frontal alpha is lower. It is a within-participant question, and the answer is yes. The change-score analysis asks whether the participants who desynchronised the most are also the participants who gained the most balance overall. It is a between-participant question, and the answer is unclear.

The second question is the one that matters for whether alpha modulation identifies who benefits, so we treat the evidence for a specific contribution of alpha modulation to balance as weak. With n=20, a ceiling on the BBS-3P (Table 3), and no control group, such a contribution cannot be separated from broader working memory gains, practice effects, or non-specific engagement with the intervention. Both analyses are reported as hypothesis-generating.

#### 3.7.2. Theta Power During N-Back Training

P3-associated frontal theta during the N-back working memory task showed more coherent neuro–behavioural coupling than alpha (Table 6). Here, theta power was quantified within the P3-locked analysis window and averaged across stages, respectively. Left- and right-frontal coefficients were of comparable magnitude in every cell, so these data provide no evidence of a lateralised theta–behaviour relationship; no lateralisation index was pre-specified or computed.

The theta–behaviour associations were not uniform across outcomes, and the comparison between the two columns of Table 6 is informative. Only the association with CTMT-A was retained essentially unchanged when moving from stage-pooled to participant-level modelling (left-frontal ρ=−0.58 pooled, $r_{\mathrm{rm}}=-0.53$, p<0.001), indicating that participants who engaged frontal theta more strongly during working memory demands also completed the visual-scanning task faster. The association with BBS-3P was smaller but retained (left-frontal $r_{\mathrm{rm}}=+0.37$, p=0.018).

By contrast, the associations with CTMT-B and DGI-4 were not retained under participant-level modelling. The pooled coefficient for left-frontal theta and CTMT-B (ρ=−0.37, p=0.003) fell to $r_{\mathrm{rm}}=-0.19$ (p=0.235) once repeated observations within participants were modelled, and the corresponding DGI-4 association fell to $r_{\mathrm{rm}}=+0.24$ (p=0.135). Stage-pooled correlations treat each participant’s three stage observations as independent and thereby inflate the effective sample size from 20 to 60; the attenuation seen here is the expected consequence of removing that inflation. We therefore state plainly that the participant-level evidence for theta coupling to set-shifting performance and to gait adaptability is weak. What the analysis does isolate is a specific and comparatively robust pairing: frontal theta with processing speed, present in both hemispheres at similar magnitude ($r_{\mathrm{rm}}=-0.53$ left, −0.51 right), together with a smaller but retained link to balance. This selectivity is itself informative, since a feature that tracks one cognitive domain and not another is more useful as a training target than one that correlates indiscriminately. These observations are compatible with the established role of frontal midline theta in working memory maintenance and performance monitoring, although the present design cannot establish that the training caused either the oscillatory change or the behavioural change.

To test whether these neuro–behavioural associations were driven by individual differences in neurofeedback performance, we repeated the analysis in the subset of participants with valid NF car–game recordings (n=20) and included the NF forward ratio as a covariate (Table 8). The forward ratio indexed NF performance as the proportion of game events in which the car moved forward (successful alpha up-regulation above the upper threshold) relative to all movement events (forward + backward).

The NF forward ratio showed only weak associations with the outcome measures, the largest being a negative association with BBS-3P that did not reach the nominal threshold (ρ=−0.39, p=0.091; Table 8). Partial correlations between left-frontal theta and each outcome, controlling for the forward ratio, were similar in magnitude to the corresponding participant-level coefficients and likewise did not reach the nominal threshold (all p≥0.104). This suggests that the theta–behaviour associations reported in Table 6 are not simply an artefact of individual differences in NF car–game performance. It does not, however, establish the converse: with n=20 these partial correlations are imprecisely estimated, and the analysis has limited power to detect a modest confounding effect even if one is present.

#### Scope of the Correlation Analyses

The analysis screens candidates rather than confirming them, and it discriminates between them. Four cells showed moderate-to-large participant-level coefficients and were retained as candidate associations: right-frontal alpha with BBS-3P (r=−0.64) and DGI-4 (r=−0.48) and left- and right-frontal theta with CTMT-A (r=−0.53 and −0.51). Three that appeared convincing when pooled were not retained, most clearly left-frontal theta with CTMT-B (ρ=−0.37 pooled versus r=−0.19 at participant level). Section 4.4 sets out how this shortlist should be used.

## 4. Discussion

### 4.1. Feasibility of the BCI Architecture

Adherence was 100% across 24 sessions, and no session was terminated early, indicating that the programme is deliverable in a supervised experimental setting in community-dwelling older adults. Repetitive cognitive training is typically susceptible to attrition, and the gamified interface appears to have sustained engagement; user-feedback ratings were correspondingly high. Feedback was delivered with an end-to-end latency below 250 ms, within the window required for operant conditioning [20], which indicates that this consumer-grade EEG system was able to meet the timing requirements of the present closed-loop neurofeedback implementation. No severe adverse events occurred. Visual fatigue was reported by 55% of participants and represents an important tolerability consideration; whether this resulted from sustained visual attention, the gamified display, session duration, or another aspect of the protocol cannot be determined from the present design. Feasibility, the primary outcome of this study, is therefore established.

### 4.2. Alpha Dynamics: Gating at the Trained Site, Engagement Elsewhere

The two frontal regions moved in opposite directions across the programme. Left-frontal alpha, the site at which the neurofeedback contingency reinforced up-regulation, rose from a median of 0.13 to 2.00 dB, and the Early-to-Later increase survived correction for multiple comparisons. Right-frontal alpha fell over the same period, from 0.72 to −0.94 dB, and that decrease also survived correction.

Within the inhibition-timing framework these are not competing observations but the two halves of the same mechanism. Alpha synchronisation is held to index the active suppression of task-irrelevant cortical processing [28,30], whereas alpha desynchronisation indexes engagement of the underlying cortex [29]. The left-frontal increase is the outcome the intervention was designed to produce. Alpha rose at the site the neurofeedback reinforced, in the direction it reinforced, and the Early-to-Later increase survived correction for multiple comparisons. This was the single oscillatory prediction specified before the data were analysed, and it was met. What an uncontrolled design cannot establish is that the neurofeedback caused the change rather than accompanying it; the observation is that participants ended the programme with higher alpha at the trained site than they began it. The right-frontal decrease, by contrast, was neither trained nor anticipated, and indexes increased engagement of a region that was not the target.

The behavioural pattern is consistent with that reading and converges with two independent studies. Right-frontal alpha showed the largest participant-level association in the dataset with balance (r=−0.64) and a substantial one with gait adaptability (r=−0.48): at the stages where a participant’s right-frontal alpha was most desynchronised, their balance and gait scores were highest.

At the behavioural level, visuospatial attention is an established determinant of postural stability in this population. A recent systematic review of eye-tracking and balance studies in adults over 60 concluded that visual attention measures are consistently associated with posture, balance, and gait outcomes and characterised visual attention as a central and modifiable determinant of postural stability [59]; poorer visuospatial ability is likewise associated with greater risk of fall. Right frontal and parietal networks are the networks implicated in visuospatial attention and the monitoring of external space [60,61,62,63], so a link between right-frontal engagement and postural performance is expected rather than novel.

At the electrophysiological level, the direction we observe has been reported before at comparable spatial resolution. In 30 community-dwelling older adults performing dual-task standing, Kahya and colleagues found that greater alpha power over anterior-right and central-right regions was associated with greater postural sway velocity, that is, with poorer postural control [64]. Lower right-anterior alpha accompanying better postural performance is therefore the same relationship we report, in the same population and in the same direction, obtained without source localisation.

A further consideration is the adaptive threshold. Because the trigger tracked each participant’s own running baseline, success was defined relative to a moving criterion rather than an absolute one. This cuts both ways. A rise in absolute alpha does not mechanically generate more successful trials, since the threshold rises with it, and the reported outcome, alpha power in dB relative to a pre-event baseline, is computed independently of the threshold. However, the threshold determines which epochs are marked as event 6 and therefore enter the analysis; this event selection is a potential source of circularity and conditioning bias, and a shift in the measured alpha modulation could in principle reflect a change in the selection criterion rather than improved regulation. Separating genuine regulation from adaptation to the thresholding mechanism would require a fixed-threshold or yoked-sham condition, which the present design does not include.

Three further qualifications apply. The association is correlational and may reflect a third factor, such as general engagement with the programme, acting on both. It was identified among sixteen candidate associations without correction. With fourteen electrodes, “right-frontal” denotes the scalp sites at which component activity was strongest rather than an identified cortical source. We therefore do not claim a dual-action mechanism in which left-hemisphere gating releases resources for right-hemisphere monitoring; that account is compatible with these data but cannot be distinguished from alpha drifting in opposite directions at the two sites for unrelated reasons.

### 4.3. Frontal Theta and Working Memory Engagement

The N-back behavioural trajectories exhibit significant gains at moderate difficulty (1-back to 3-back), followed by a performance plateau at higher loads (4-back to 6-back). From the perspective of functional plasticity, this stabilisation is consistent with the hypothesis that cortical activity scales with working memory load before potentially reaching a compensatory ceiling [65].

The longitudinal modulation of P3-associated frontal theta power identifies a candidate neurophysiological feature for evaluation in a controlled cognitive-training study. The pronounced theta enhancement in the frontal region observed during the middle phase likely reflects the increased expenditure of cognitive effort and the deployment of proactive control mechanisms as participants adapted to escalating N-back task demands [47,66]. This peak in recruitment, followed by stabilisation in the later stage, is compatible with a transition toward more efficient recruitment—a pattern compatible with learning-related change, although familiarisation and other time-dependent effects cannot be excluded.

The evidence that frontal theta indexes performance across functional domains is more selective than the stage-pooled correlations initially suggested (Table 6), and the pattern of what survived is itself informative. Once repeated observations within participants were modelled rather than pooled, the association with processing speed (CTMT-A) remained clearly supported in both hemispheres (r=−0.53 left, −0.51 right) and the association with balance was retained at a smaller magnitude. The associations with cognitive flexibility (CTMT-B) and gait adaptability (DGI-4) fell well below the nominal threshold.

This dissociation between processing speed and cognitive flexibility is not an anomaly of our sample. Darna and colleagues [46] compared younger and older adults on a set-shifting task and found that whereas younger participants showed midfrontal theta power scaling with shift difficulty, older participants showed lower theta overall and no task-related modulation with shifting demand. If frontal theta in older adults does not track set-shifting load, then a weak theta–CTMT-B association is the expected result rather than a failure of the present analysis. The same literature reports blunted theta modulation with memory load in ageing [39,40], which makes the increase in frontal theta observed here over the training period notable in this population, though the uncontrolled design cannot attribute it to the intervention.

Left- and right-frontal theta coefficients were of similar size throughout. Rather than being a null result, this bilateral pattern is the one anticipated for this population: the HAROLD model describes precisely such a reduction in hemispheric asymmetry in older adults [42], de-differentiation describes the accompanying loss of functional specificity, as established by neuroimaging evidence of age-related neural dedifferentiation [48,67], and expanded, bilaterally symmetric recruitment has been reported in this population in the sensorimotor domain [43]. Frontal theta in this cohort therefore behaves as a bilateral executive index, and any future protocol using it as a training target should extract it bilaterally rather than assume a left-lateralised generator. The alpha findings show the opposite profile, remaining clearly asymmetric because one hemisphere was trained, so the two rhythms should not be treated as a single lateralised mechanism.

The surviving associations are compatible with the proposal that frontal theta indexes a shared executive resource drawn on by both working memory manipulation and complex motor coordination [44,68,69], and partial correlations controlling for the NF car forward ratio were of similar magnitude, indicating that they are not merely a reflection of neurofeedback task performance. They remain cross-sectional associations observed in an uncontrolled cohort of 20 participants and cannot establish that frontal theta is a marker of training-related change, but they do identify it as a feature worth carrying into a design that can.

### 4.4. Selecting Features for Future Fall-Prevention BCI

Translation of EEG-based BCI to fall prevention is currently limited by methodological heterogeneity and by weak evidence that cognitive gains reach physical function [18]. A single-arm study cannot supply that evidence. Its contribution is to narrow the set of candidate features for a subsequent trial.

Two of the three expectations set out in the Introduction were met, and the third was not. Participants did acquire control of the trained signal: left-frontal alpha rose in the direction the neurofeedback reinforced, and the concurrent right-frontal decrease is interpretable as engagement rather than as a failure of training. Frontal theta did increase under adaptive working memory load, and did so bilaterally, as expected in a population whose frontal recruitment is characteristically less lateralised. The expectation that executive gains would be accompanied by functional ones is only partly borne out. Associations of the anticipated sign were present for right-frontal alpha with balance and gait and for frontal theta with processing speed, but the behavioural changes to which they refer were small: BBS-3P and DGI-4 scores rose, yet no participant’s gait change reached the minimal detectable change in the DGI-4, and both scales were near ceiling by the Later stage. Cognitive–motor coupling therefore remains a proposition for a future trial rather than a finding of this one.

Four of sixteen band × region × outcome combinations retained moderate-to-large coefficients under participant-level modelling: right-frontal alpha with balance and with gait adaptability and bilateral frontal theta with processing speed. Several that appeared convincing under stage-pooled analysis did not. The two rhythms also behaved differently, theta as a bilateral executive index and alpha as an asymmetric one, and should not be treated interchangeably.

This has a methodological implication beyond the present dataset. Stage-pooled correlation would have reported the theta–CTMT-B association as significant (ρ=−0.37, p=0.003) where the participant-level estimate is r=−0.19 (p=0.235). Longitudinal neurofeedback studies routinely pool across sessions in this way, so some of the heterogeneity in this literature [18] may be analytic rather than interventional. We recommend repeated-measures correlation or mixed-effects modelling as the default.

Three further steps are required before any of these features could be used as a biomarker. First, a comparison arm: methodological consensus in the field treats this as essential rather than optional [70], and randomisation to active training, an alternative band, or sham with participants and assessors masked [71] would separate feature-specific effects from the experience of training. Second, a population and instruments with room to change: our cohort was healthy and near ceiling, whereas trials designed to demonstrate functional benefit recruit older adults with measurable balance or mobility impairment [72], and success should be judged against the change an instrument can detect [53]. Third, evidence that the feature is stable when nothing has changed, as established for frontal alpha in older adults [73]; no equivalent reliability evidence exists for the component-level features used here. Anatomical description requires a denser montage, since fourteen electrodes cannot identify cortical sources [74].

The present study contributes the step preceding these: an interval-estimated shortlist of candidate features and the effect sizes on which a future trial can be powered.

### 4.5. Limitations and Future Directions

This study is subject to several methodological limitations that warrant consideration:

- Sample Size and Design: This was a single-arm feasibility study with a modest sample size (n=20) and no parallel control group. Consequently, whilst within-subject improvements were significant, we cannot definitively rule out placebo effects or spontaneous regression to the mean.
- Hardware Constraints: The use of a consumer-grade, 14-channel EEG headset limits spatial resolution. Source localisation and specific claims regarding anatomical generators (e.g., differentiating specific prefrontal sub-regions) must be interpreted with caution due to volume conduction effects [74].
- Component Selection: Although selection criteria were defined in advance and applied blind to training stage and outcome, the final judgement of whether a component met them was made by a single rater. Inter-rater reliability was not assessed, so the reproducibility of the selection step is unquantified. Establishing it is a prerequisite before any of these features could be treated as a validated marker.
- Longitudinal Follow-up: This study assessed outcomes immediately post-intervention (Week 8). The long-term retention of these cognitive and motor gains remains to be established in future follow-up studies.
- Practice Effects: Improvements in the N-back task and cognitive assessments (CTMT-A/B) may partially reflect task-specific practice effects. Future pivotal trials should incorporate active control groups (e.g., sham neurofeedback) to isolate specific neuroplastic effects.
- Survivorship Bias: The attrition of participants at higher N-back difficulty levels (5-back and 6-back) introduces potential survivorship bias, as data at these loads reflect only the highest-performing individuals.

## 5. Conclusions

This feasibility study shows that a gamified programme combining alpha neurofeedback with an adaptive N-back task can be delivered in a supervised experimental setting to community-dwelling older adults over 24 sessions. Adherence was complete, the protocol was well tolerated, and usable EEG was obtained at each stage, which establishes the primary feasibility outcome.

Changes were observed in both targeted oscillatory features over the training period. Left-frontal alpha increased at the site the neurofeedback reinforced, frontal theta increased bilaterally under working memory load, and right-frontal alpha decreased. Among sixteen candidate EEG–behaviour associations, four showed moderate-to-large participant-level coefficients and were retained as candidate associations: right-frontal alpha with balance and gait adaptability and bilateral frontal theta with processing speed.

These associations are exploratory. The design is uncontrolled, the analyses were not corrected for multiplicity, and the functional changes did not exceed the minimal detectable change in the instruments used, so this study does not establish that the intervention improved balance or gait nor that any oscillatory feature is a validated marker of such improvement.

Within those bounds, this study makes three contributions to the field. It demonstrates that consumer-grade EEG hardware can deliver a closed-loop, gamified neurofeedback programme to community-dwelling older adults at a dose of 24 sessions over eight weeks, with complete adherence, no severe adverse events, and sustained engagement. It provides a participant-level, repeated-measures characterisation of how frontal alpha and theta behave over an extended combined training programme in this population. The two rhythms follow distinct spatial profiles: alpha is asymmetric around the trained site, whereas theta is bilateral. The same comparison shows that stage-pooled analysis, the default in this literature, can misstate which EEG–behaviour associations the data support. Finally, it delivers what a subsequent trial needs most: a shortlist of candidate features, together with the effect-size estimates required to power an adequately sized, sham-controlled trial. Right-frontal alpha is nominated for balance and gait adaptability, and bilateral frontal theta is nominated for processing speed. These findings support evaluation of the platform in an appropriately powered, controlled trial. A sufficiently powered, sham-controlled study in older adults with measurable risk of fall, using outcome measures with adequate headroom and alternate cognitive-test forms, is required to determine whether the observed neurophysiological changes are intervention-specific and translate into clinically meaningful functional benefit.

## Figures and Tables

**Table 1:** Baseline participant characteristics (n=20). Demographics are mean ± SD; outcome measures are median [IQR].

Characteristic	Value
Age (years)	65.0±3.3
Sex (female/male)	13/7
Handedness	20 right-handed
MMSE (max. 30)	29.2±0.5
BBS-3P (max. 28)	26.0 [26.0, 27.0]
at ceiling	2/20 (10%)
DGI-4 (max. 12)	10.5 [10.0, 12.0]
at ceiling	6/20 (30%)
CTMT-A (s)	96.0 [78.5, 108.3]
CTMT-B (s)	98.0 [87.0, 113.8]

**Table 2:** Maximum achieved N-back level as an index of working memory capacity.

N-Back Level	Number of Participants Able to Complete Level
1–3	20
4	15
5	4
6	1

**Table 3:** Behavioural outcomes by training stage, median [IQR] (n=20). Stages are defined by session number: Early, sessions 1–8; Middle, sessions 9–16; Later, sessions 17–24. Lower CTMT-A/B times indicate better performance. Lower panel: participants scoring at the ceiling of each scale.

Outcome	Early	Middle	Later
BBS-3P (max. 28)	26.0 [26.0, 27.0]	27.0 [26.8, 28.0]	28.0 [27.0, 28.0]
DGI-4 (max. 12)	10.5 [10.0, 12.0]	11.0 [11.0, 12.0]	12.0 [11.8, 12.0]
CTMT-A (s)	96.0 [78.5, 108.3]	59.5 [52.0, 63.3]	43.0 [39.5, 55.0]
CTMT-B (s)	98.0 [87.0, 113.8]	89.5 [77.3, 98.8]	78.5 [63.8, 92.3]
*Participants at ceiling, n (%)*			
BBS-3P	2 (10%)	8 (40%)	13 (65%)
DGI-4	6 (30%)	7 (35%)	15 (75%)

**Table 4:** Stage contrasts for behavioural outcomes (n=20). Matched-pairs rank-biserial *r* with 95% BCa confidence intervals; positive values indicate improvement. “*n* changed” counts participants whose score differed between the two stages. Wilcoxon signed-rank *p*, uncorrected.

Outcome	Contrast	*n* Changed	*r* [95% CI]	*p*
BBS-3P	Early vs. Middle	14	+0.92 [+0.50, +1.00]	0.002
	Middle vs. Later	11	+0.59 [−0.09, +0.92]	0.078
	Early vs. Later	17	+0.90 [+0.56, +1.00]	0.001
DGI-4	Early vs. Middle	10	+0.67 [+0.00, +1.00]	0.052
	Middle vs. Later	10	+1.00 [+1.00, +1.00]	0.004
	Early vs. Later	13	+1.00 [+1.00, +1.00]	0.001
CTMT-A	Early vs. Middle	20	+0.99 [+0.73, +1.00]	<0.001
	Middle vs. Later	20	+1.00 [+1.00, +1.00]	<0.001
	Early vs. Later	20	+1.00 [+1.00, +1.00]	<0.001
CTMT-B	Early vs. Middle	20	+0.75 [+0.27, +0.94]	0.003
	Middle vs. Later	20	+0.76 [+0.25, +0.96]	0.003
	Early vs. Later	20	+0.99 [+0.80, +1.00]	<0.001

**Table 5:** Frontal alpha power during neurofeedback against behavioural outcomes (n=20; 60 observations). Negative alpha values indicate greater desynchronisation. *p* refers to the rmcorr coefficient and is uncorrected.

Region	Behaviour	Stage-Pooled ρ [95% CI]	Rmcorr *r*	*p*
Left-Frontal	CTMT-A (s)	−0.37 [−0.57, −0.09]	−0.41	0.008
Left-Frontal	CTMT-B (s)	−0.17 [−0.46, +0.14]	−0.24	0.138
Left-Frontal	BBS-3P	+0.19 [−0.11, +0.44]	+0.27	0.084
Left-Frontal	DGI-4	+0.37 [+0.09, +0.61]	+0.42	0.006
Right-Frontal	CTMT-A (s)	+0.31 [−0.11, +0.53]	+0.47	0.002
Right-Frontal	CTMT-B (s)	+0.08 [−0.25, +0.37]	+0.31	0.045
Right-Frontal	BBS-3P	−0.31 [−0.51, −0.02]	−0.64	<0.001
Right-Frontal	DGI-4	−0.31 [−0.54, −0.02]	−0.48	0.001

**Table 6:** P3-associated frontal theta power during the N-back task against behavioural outcomes (n=20; 60 observations). Lower CTMT-A/B times indicate better performance. *p* refers to the rmcorr coefficient and is uncorrected.

Region	Behaviour	Stage-Pooled ρ [95% CI]	Rmcorr *r*	*p*
Left-Frontal	CTMT-A (s)	−0.58 [−0.76, −0.41]	−0.53	<0.001
Left-Frontal	CTMT-B (s)	−0.37 [−0.64, −0.09]	−0.19	0.235
Left-Frontal	BBS-3P	+0.29 [+0.06, +0.49]	+0.37	0.018
Left-Frontal	DGI-4	+0.24 [+0.00, +0.46]	+0.24	0.135
Right-Frontal	CTMT-A (s)	−0.50 [−0.71, −0.27]	−0.51	<0.001
Right-Frontal	CTMT-B (s)	−0.20 [−0.52, +0.14]	−0.14	0.396
Right-Frontal	BBS-3P	+0.36 [+0.05, +0.56]	+0.41	0.008
Right-Frontal	DGI-4	+0.24 [−0.03, +0.48]	+0.31	0.046

**Table 7:** Correlation with change in BBS-3P, computed on each participant’s Early-to-Later change scores (n=20). *p*-values are uncorrected.

Change in	Correlation	ρ	*p*
Right-Frontal alpha	Spearman	−0.40	0.170
N-back accuracy	Spearman	−0.52	0.067
Right-Frontal alpha	Partial ^*a*^	−0.32	0.310

^*a*^ Controlling for change in N-back accuracy.

**Table 8:** Participant-level Spearman correlations involving the neurofeedback (NF) forward ratio (n=20). Lower panel: left-frontal theta against each outcome, controlling for the forward ratio. *p*-values are uncorrected.

Predictor	Behaviour	ρ	*p*
NF forward ratio	CTMT-A (s)	+0.19	0.415
	CTMT-B (s)	−0.07	0.782
	DGI-4	−0.22	0.359
	BBS-3P	−0.39	0.091
*Controlling for NF forward ratio*			
Left-Frontal theta	CTMT-A (s)	−0.39	0.104
	CTMT-B (s)	−0.16	0.508
	DGI-4	+0.09	0.720
	BBS-3P	+0.05	0.826

## Data Availability

The data presented in this study are available upon request to the corresponding author. The data are not publicly available due to privacy reasons.

## Abbreviations

The following abbreviations are used in this manuscript:

ACC	Accuracy
BCI	Brain–Computer Interface
BBS-3P	Berg Balance Scale, short form
CTMT-A/B	Colour Trail Making Test A / B
DGI-4	Dynamic Gait Index, 4-item
EEG	Electroencephalography
ERP	Event-Related Potential
ERSP	Event-Related Spectral Perturbation
ERS	Event-Related Synchronisation
ERD	Event-Related Desynchronisation
FDR	False Discovery Rate
FIR	Finite Impulse Response
HAROLD	Hemispheric Asymmetry Reduction in Older Adults
ICA	Independent Component Analysis
IC	Independent Component
MMSE	Mini-Mental State Examination
NF	Neurofeedback
ROI	Region of Interest
WM	Working Memory
